# Supplementary material for: Adaptive RSK‐EphA2‐GPRC5A signaling switch triggers chemotherapy resistance in ovarian cancer
Source: EMBO Mol Med. 2020 Mar 2;12(4):e11177. doi: 10.15252/emmm.201911177 (PMC7136956; doi:10.15252/emmm.201911177)
Supplement: Supplementary file 1 — Appendix [file EMMM-12-e11177-s001.pdf]

APPENDIX

Adaptive RSK-EphA2-GPRC5A signaling switch triggers chemotherapy resistance in ovarian cancer

Lidia Moyano-Galceran, Elina A Pietilä, S Pauliina Turunen, Sara Corvigno, Elisabet Hjerpe, Daria Bulanova, Ulrika Joneborg, Twana Alkasalias, Yuichiro Miki, Masakazu Yashiro, Anastasiya Chernenko, Joonas Jukonen, Madhurendra Singh, Hanna Dahlstrand, Joseph W Carlson, Kaisa Lehti

Table of Contents

Appendix Figure S1: Characterization of HGSC cell lines.

Appendix Figure S2: Cisplatin treatment induces EphA2 (total and pS897) in patient-derived HGSC cells *ex vivo*.

Appendix Figure S3: *MEK-ERK1/2 pathway inhibition does not increase cell sensitivity to platinum-based treatment.*

Appendix Figure S4: RSKi prevents the platinum-induced EphA2-GPRC5A co-localization in OC cells.

Appendix Figure S5: EphA2 and GPRC5A are mutually negatively regulated in platinum-sensitive cells.

Appendix Figure S6: RSKi combinatorial treatment induces apoptosis in 3D co-culture.

Appendix Figure S7: RSKi reverts platinum-induced EphA2 phosphorylation switch in patient-derived HGSC cells but does not alter the tumor-suppressive EphA2-pY588 in normal fibroblasts.

Appendix Table S1: Characteristics of HGSC patients included in the TMA.

Appendix Table S2: Correlation analysis of maximum intensity of GPRC5A staining between primary and metastatic tumor sites.

Appendix Table S3: Correlation analysis between maximum intensity of GPRC5A staining and the clinico-pathological variables.

Appendix Table S4: Survival analysis in primary versus metastatic tumors.

Appendix Table S5: Univariate and multivariate analysis for the association of OS to the clinico-pathological variables and maximum intensity of GPRC5A staining.

Appendix Table S6: Univariate and multivariate analysis for the association of PFS to the clinico-pathological variables and maximum intensity of GPRC5A staining.

Appendix Table S7: Correlation of ORR and treatment sensitivity with maximum intensity of GPRC5A staining.

Appendix Table S8: Probe IDs from The Cancer Cell Line Encyclopedia.

Appendix Table S9: siRNA sequences.

Appendix Table S10: Exact significant p-values in figures.

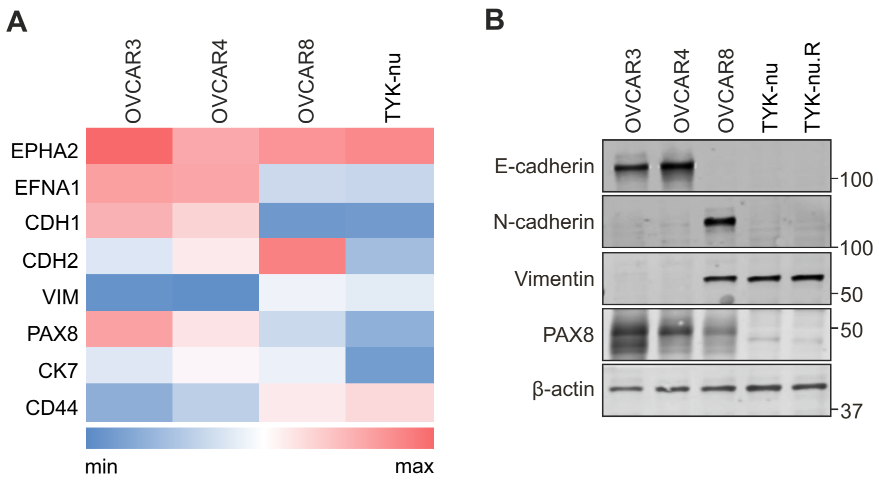
A
OVCAR3
OVCAR4
OVCAR8
TYK-nu
EPHA2
EFNA1
CDH1
CDH2
VIM
PAX8
CK7
CD44
min
max
B
OVCAR3
OVCAR4
OVCAR8
TYK-nu
TYK-nu.R
E-cadherin
N-cadherin
Vimentin
PAX8
β-actin
100
100
50
50
37

Appendix Figure S1.

A Heat map for EphA2, ephrinA1 (EFNA1), E-cadherin (CDH1), N-cadherin (CDH2), vimentin (VIM), PAX8, cytokeratin 7 (CK7) and CD44 mRNA expression in OC cell lines. EphA2 expression was high in all cells, whereas expression of the ligand ephrinA1 was high in OVCAR3 and OVCAR4, but low in OVCAR8 and TYK-nu. Data obtained from the Cancer Cell Line Encyclopedia (https://portals.broadinstitute.org/ccle); no data available for TYK-nu.R. See Appendix Table S8 for probe IDs.

B E-cadherin, N-cadherin, vimentin and PAX8 protein expression in OC cell lines. High expression of E-cadherin and low vimentin in OVCAR3 and OVCAR4 defined an epithelial phenotype, whereas the opposite pattern was seen in the more mesenchymal OVCAR8, TYK-nu and TYK-nu.R, with low E-cadherin and high vimentin expression. The same β-actin detection for these samples is shown in Fig 2C.

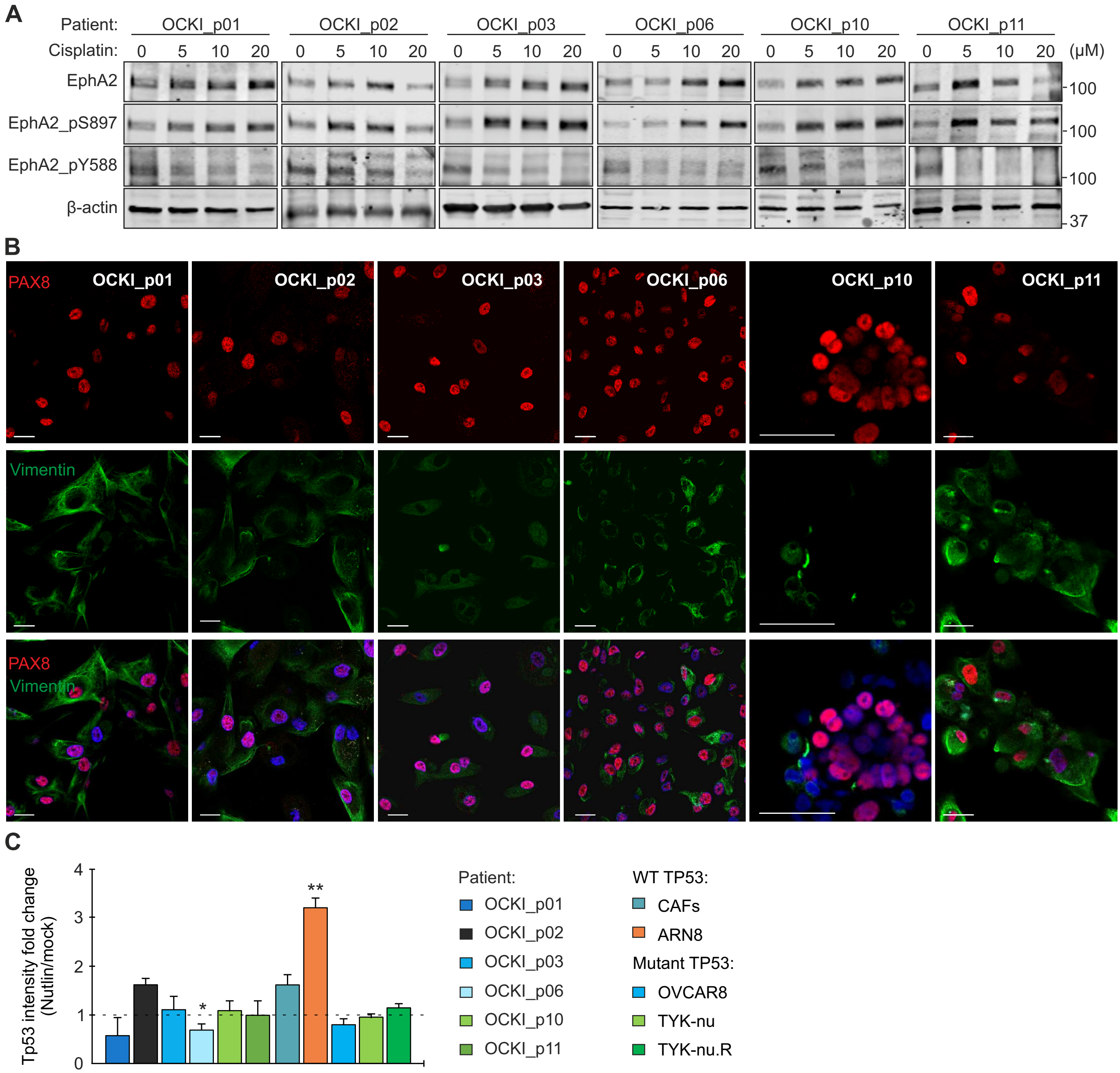
A
Patient: OCKI_p01 OCKI_p02 OCKI_p03 OCKI_p06 OCKI_p10 OCKI_p11
Cisplatin: 0 5 10 20 (μM)
EphA2
EphA2_pS897
EphA2_pY588
β-actin
100
100
100
37
B
PAX8
OCKI_p01
OCKI_p02
OCKI_p03
OCKI_p06
OCKI_p10
OCKI_p11
Vimentin
PAX8
Vimentin
C
Tp53 intensity fold change (Nutlin/mock)
0
1
2
3
4
*
**
Patient:
OCKI_p01
OCKI_p02
OCKI_p03
OCKI_p06
OCKI_p10
OCKI_p11
WT TP53:
CAFs
ARN8
Mutant TP53:
OVCAR8
TYK-nu
TYK-nu.R

Appendix Figure S2.

A EphA2 (total and phosphorylated) in patient-derived HGSC cells treated with 0-20 μM cisplatin for 72 h were assessed by immunoblotting. EphA2 (total and pS897) were increased upon treatment coincident with decreased EphA2-pY588.
B Confocal micrographs of PAX8 (red) and vimentin (green) in patient-derived adherent cells. Scale bars: 20 μm.
C Chart illustrates p53 fold change after nutlin treatment, quantified from confocal micrographs of p53 in patient-derived adherent cells untreated or treated with nutlin for wild-type TP53 stabilization, where mock was set to 1. Patient-derived cancer-associated fibroblasts (CAFs) and ARN8 melanoma cells (TP53 wild-type) as well as the OC cell lines OVCAR8, TYK-nu and TYK-nu.R (mutant TP53) were used as controls. p53 levels were significantly increased after treatment in ARN8 cells, whereas in patient-derived cells were not significantly increased, as seen in mutant TP53 OC cell lines. N = 3.

Data information: In (C) data are presented as mean (SD). P-values provided in Appendix Table S10, Student's t-test.

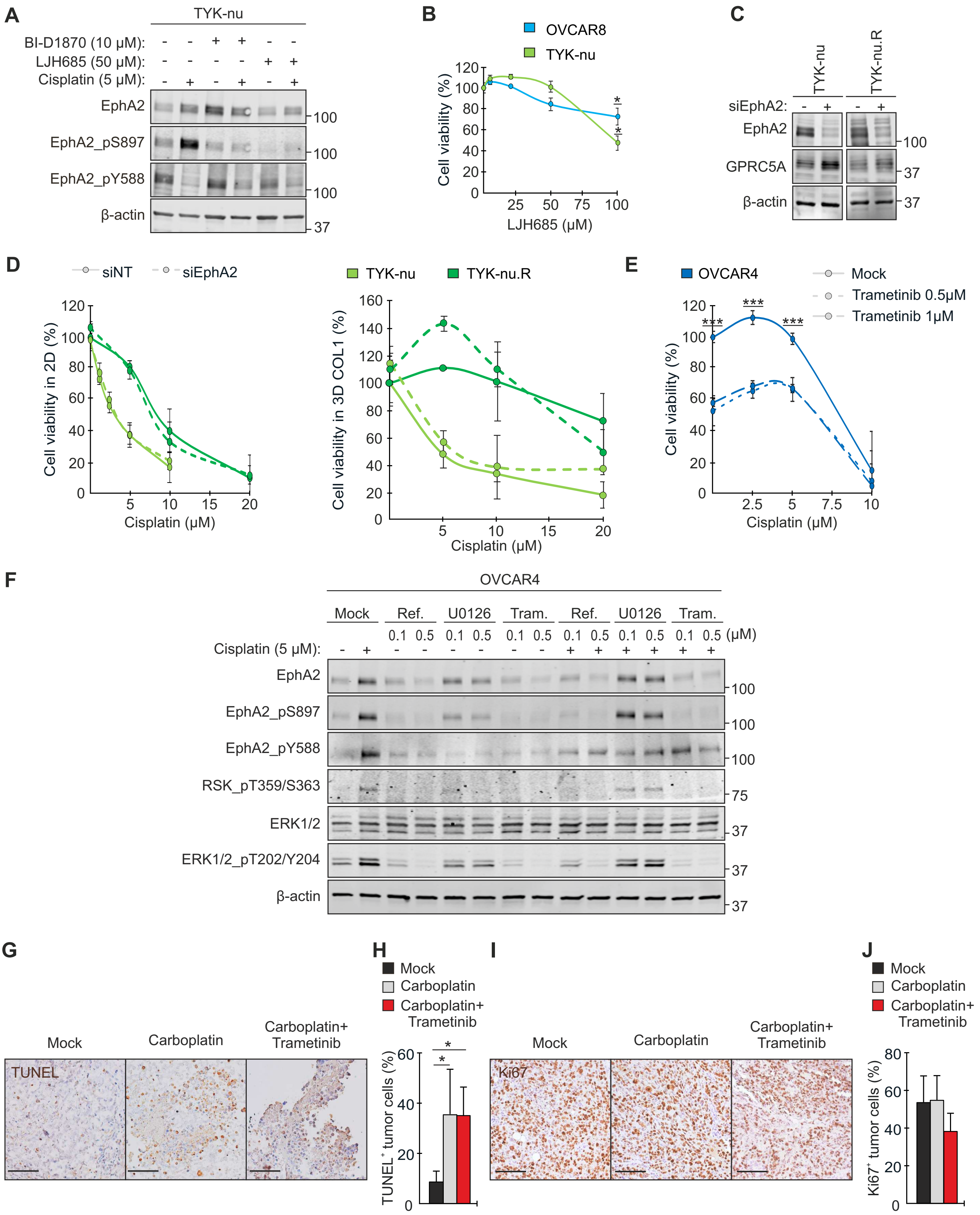

A
TYK-nu
BI-D1870 (10 μM): - - + + - -
LJH685 (50 μM): - - - - + +
Cisplatin (5 μM): - + - + - +
EphA2
EphA2_pS897
EphA2_pY588
β-actin
100
100
100
37
B
OVCAR8
TYK-nu
Cell viability (%)
LJH685 (μM)
C
TYK-nu
TYK-nu.R
siEphA2: - + - +
EphA2
GPRC5A
β-actin
D
siNT
siEphA2
Cell viability in 2D (%)
Cisplatin (μM)
TYK-nu
TYK-nu.R
Cell viability in 3D COL1 (%)
E
OVCAR4
Mock
Trametinib 0.5μM
Trametinib 1μM
Cell viability (%)
F
OVCAR4
Mock
Ref.
U0126
Tram.
(μM)
Cisplatin (5 μM):
EphA2
EphA2_pS897
EphA2_pY588
RSK_pT359/S363
ERK1/2
ERK1/2_pT202/Y204
β-actin
G
Mock
Carboplatin
Carboplatin+ Trametinib
TUNEL
H
Carboplatin+ Trametinib
TUNEL+ tumor cells (%)
I
Ki67
J
Ki67+ tumor cells (%)

Appendix Figure S3.

A EphA2 (total and phosphorylated) in TYK-nu treated with 0-5 µM cisplatin with or without 10 µM BI-D1870 or 50 µM LJH685 for 72 h. Inhibition of the oncogenic EphA2-pS897 was achieved with both inhibitors but was greater with the more specific inhibitor LJH685.

B Cell viability of OVCAR8 and TYK-nu treated with 0-100 µM LJH685 for 72 h. Concentrations up to 50 µM did not significantly affect cell viability. N = 3.

C EphA2 and GPRC5A in TYK-nu and TYK-nu.R upon EphA2 depletion were assessed by immunoblotting. GPRC5A was markedly upregulated upon EphA2 depletion in TYK-nu but not in TYK-nu.R.

D Cell viability of TYK-nu and TYK-nu.R upon EphA2 depletion and 72 h treatment with 0-20 µM cisplatin in 2D (N = 3) and 3D collagen (N = 1 experiment with 3 technical replicates). Silencing of EphA2 had no major effect on cell viability.

E Cell viability of OVCAR4 treated with cisplatin alone or in combination with 0.5-1 µM Trametinib for 72 h. N = 3. OVCAR4 cell viability was affected by trametinib treatment alone but combinatorial trametinib-cisplatin treatment had no major effect on cisplatin treatment sensitivity.

F EphA2 (total and phosphorylated), RSK-pT359/S363, ERK1/2 (total and phosphorylated) in OVCAR4 treated with 0-5 µM cisplatin with or without 0.1-0.5 µM Refametinib (Ref.), U0126 or Trametinib (Tram.) for 72 h. Inhibition of the platinum-induced EphA2-pS897 was achieved with both Refametinib and Trametinib but was greatest with Trametinib, which even restored the tumor suppressive EphA2-pY588.

G-J Immunostainings and corresponding quantifications of TUNEL and Ki67 positive cell percentage in OVCAR4 mock (N = 4), carboplatin-treated (N = 4) and carboplatin+Trametinib (N = 5) treated mice. Scale bars: 100 µm. Addition of Trametinib to carboplatin treatment further induced apoptosis. None of the treatment modalities affected cancer cell proliferation.

Data information: In (B, D, E, H and J) data are presented as mean (SD). P-values provided in Appendix Table S10, Student's t-test (B, D, E) and Mann Whitney U test (H, J).

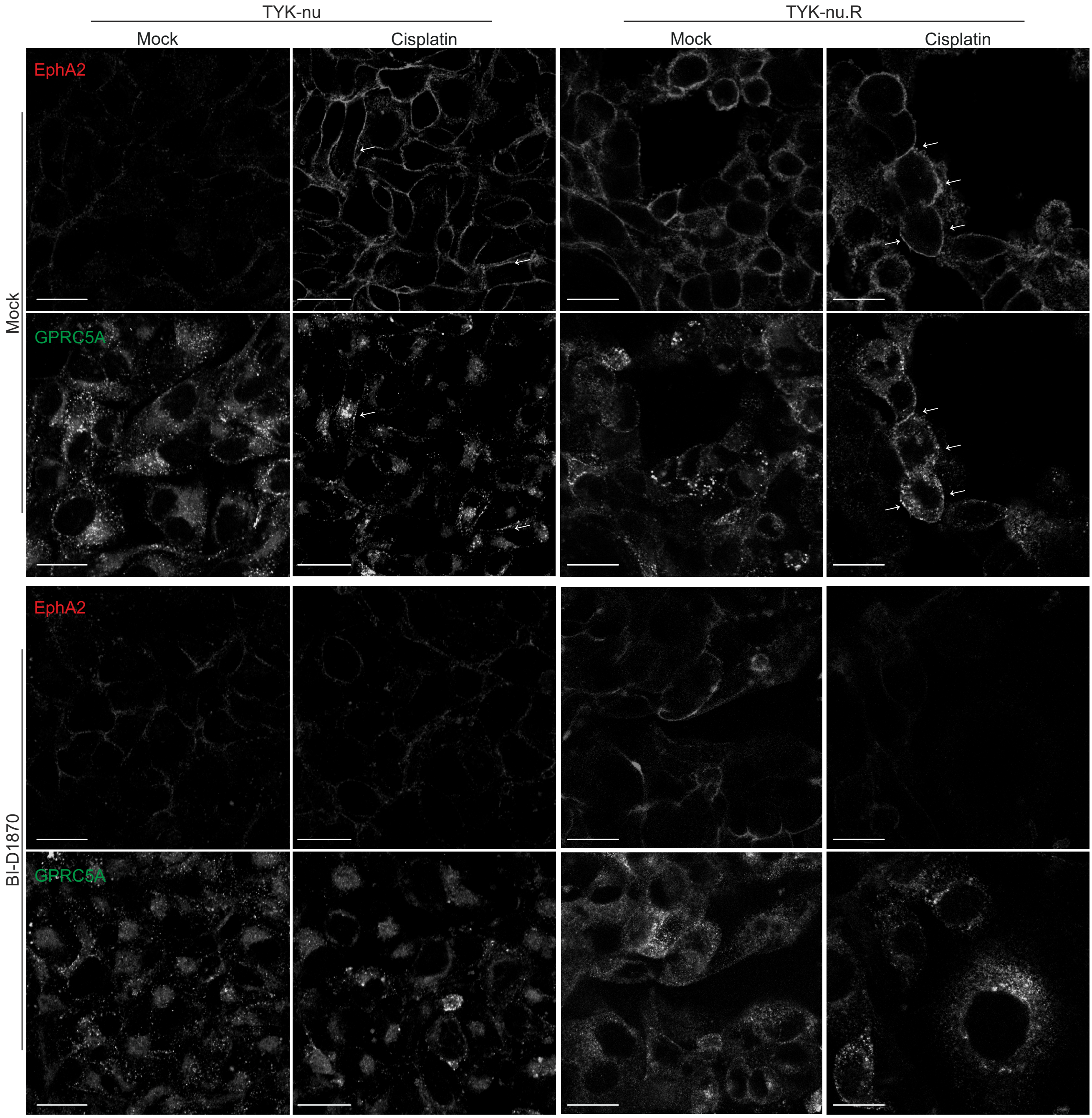
TYK-nu
TYK-nu.R
Mock
Cisplatin
Mock
Cisplatin
EphA2
GPRC5A
Mock
EphA2
GPRC5A
BI-D1870

Appendix Figure S4.

Confocal micrographs of EphA2 and GPRC5A in TYK-nu and TYK-nu.R treated with 5 μM cisplatin alone or in combination with 10 μM BI-D1870 for 72 h. Arrows point co-localization of the receptors. Scale bars: 20 μm.

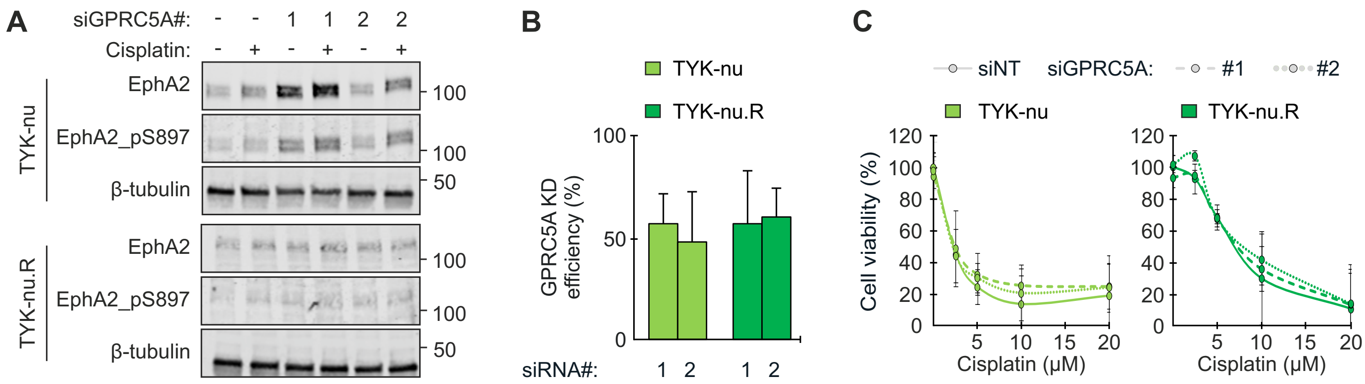
A
siGPRC5A#: - - 1 1 2 2
Cisplatin: - + - + - +
TYK-nu
EphA2
EphA2_pS897
β-tubulin
TYK-nu.R
EphA2
EphA2_pS897
β-tubulin
100
100
50
100
100
50
B
TYK-nu
TYK-nu.R
GPRC5A KD efficiency (%)
100
50
0
siRNA#: 1 2 1 2
C
siNT siGPRC5A: #1 #2
TYK-nu
TYK-nu.R
Cell viability (%)
120
100
80
60
40
20
0
5 10 15 20
Cisplatin (μM)
120
100
80
60
40
20
0
5 10 15 20
Cisplatin (μM)

Appendix Figure S5.

A EphA2 (total and pS897) in TYK-nu and TYK-nu.R upon depletion of GPRC5A were assessed by immunoblotting. Both total and EphA2-pS897 were upregulated upon silencing of GPRC5A and further upregulated upon cisplatin treatment in TYK-nu but not in TYK-nu.R.

B Chart illustrates the efficiency of siRNA-mediated GPRC5A knockdown in TYK-nu and TYK-nu.R. N = 3.

C Cell viability of TYK-nu and TYK-nu.R upon GPRC5A depletion and 72 h treatment with 0-20 µM cisplatin. N = 3. Cell viability was not affected, potentially due to low silencing efficiency.

Data information: In (B-C) data are presented as mean (SD). Student’s t-test.

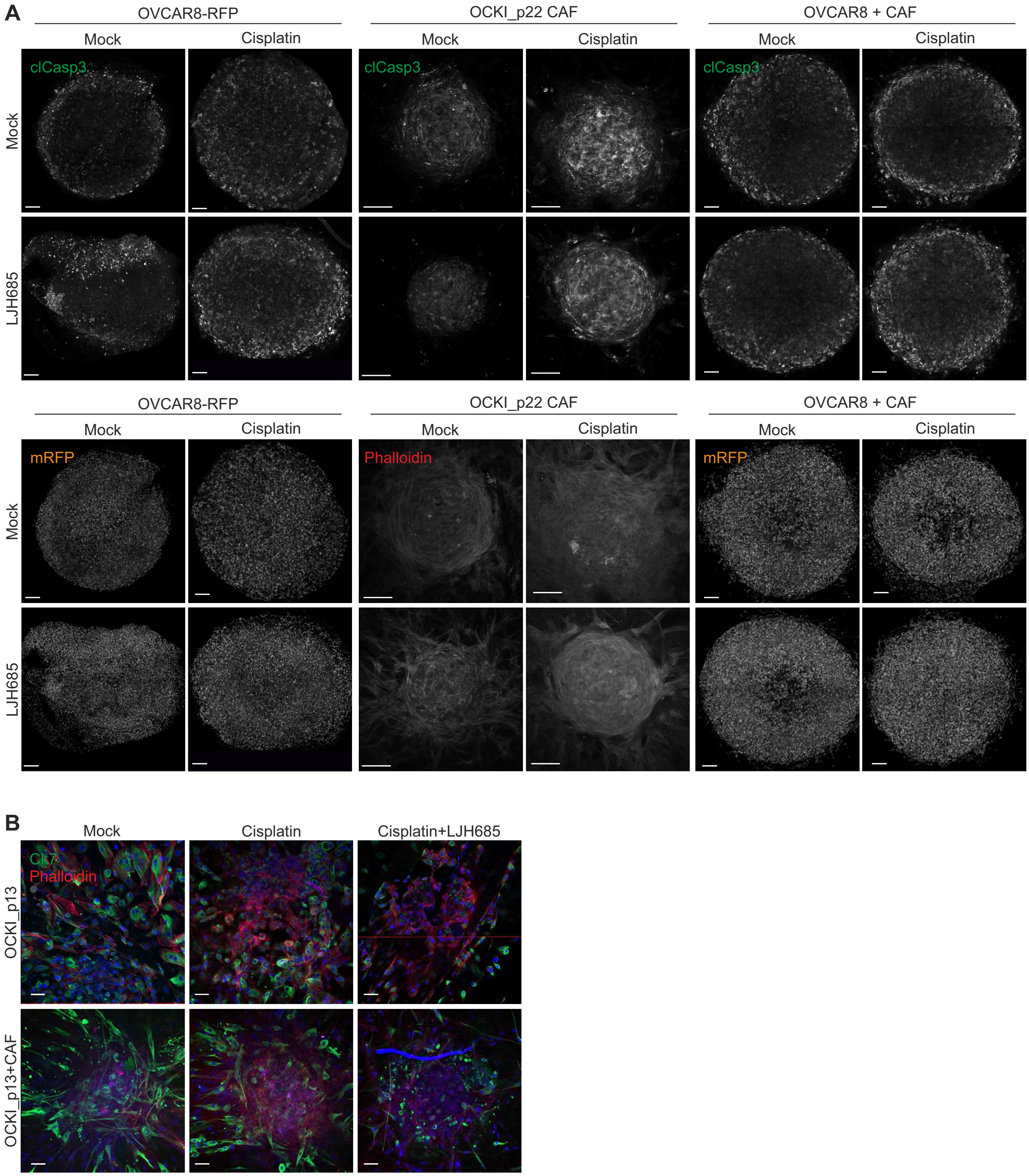
A
OVCAR8-RFP
OCKI_p22 CAF
OVCAR8 + CAF
Mock
Cisplatin
clCasp3
Mock
LJH685
mRFP
Phalloidin
B
Mock
Cisplatin
Cisplatin+LJH685
Ck7
Phalloidin
OCKI_p13
OCKI_p13+CAF

Appendix Figure S6.

A Confocal micrographs of cleaved caspase-3 (clCasp3), mRFP and phalloidin in 3D OVCAR8-RFP and OCKI_p22 CAF mono- and co-cultures treated without or with 20 µM cisplatin or 25 µM LJH685 alone or in combination for 20 h. Scale bars: 100 µm.

B Confocal micrographs show cytokeratin 7 (Ck7, green) and phalloidin (red) in 3D patient OCKI_p13 mono- and co-culture with OCKI_p22 CAF treated without or with 20 µM cisplatin or 25 µM LJH685 alone or in combination for 20 h, 4 d after 3D embedding. Scale bars: 50 µm. Red line in the micrograph for monoculture with Cisplatin+LJH685 is an artefact due to microscope acquisition, and a fiber is visible as a blue artefact in the corresponding co-culture.

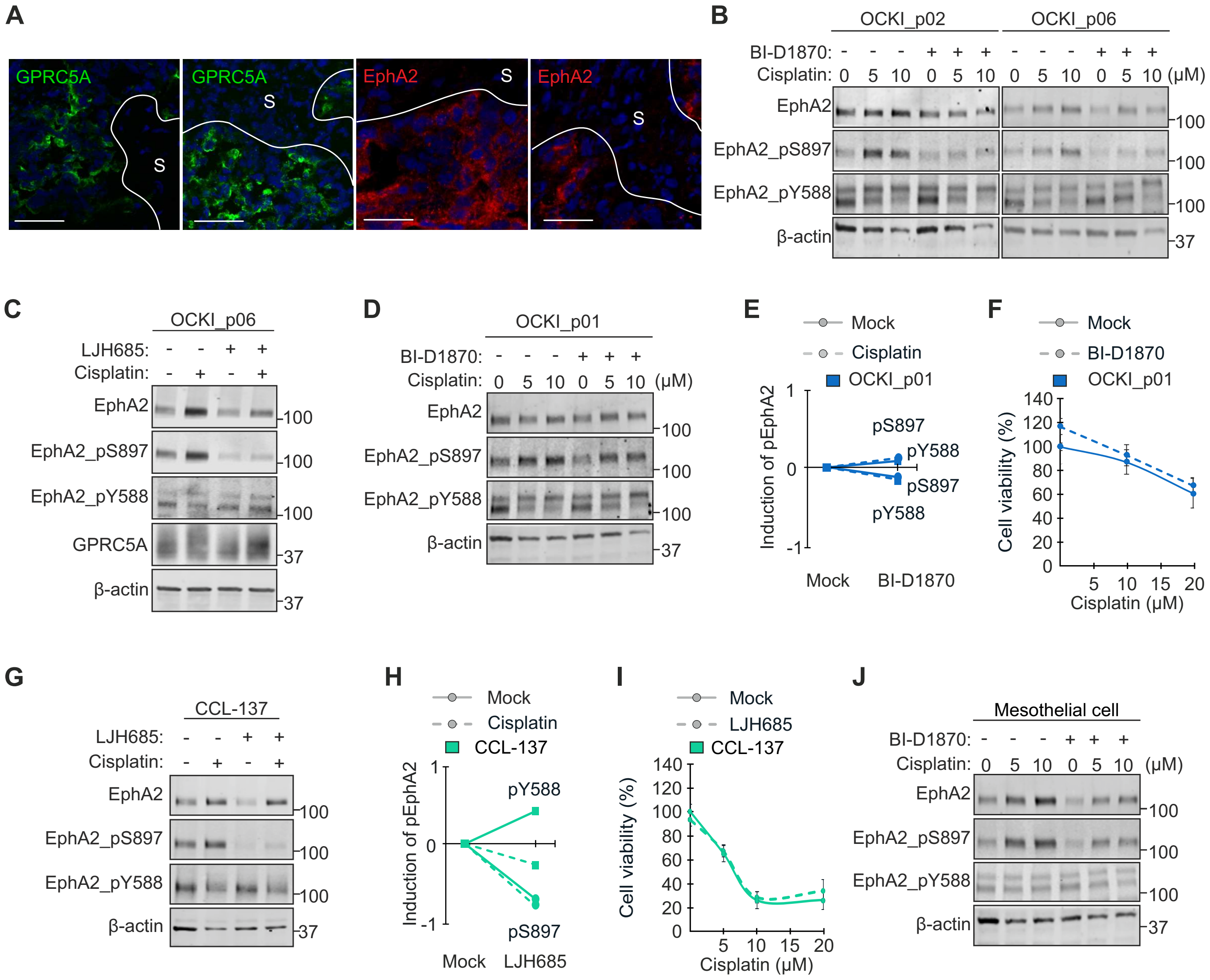

A
GPRC5A
S
GPRC5A
S
EphA2
S
EphA2
S
B
OCKI_p02
OCKI_p06
BI-D1870: - - - + + + - - - + + +
Cisplatin: 0 5 10 0 5 10 0 5 10 0 5 10 (μM)
EphA2
EphA2_pS897
EphA2_pY588
β-actin
100
100
100
37
C
OCKI_p06
LJH685: - - + +
Cisplatin: - + - +
EphA2
EphA2_pS897
EphA2_pY588
GPRC5A
β-actin
100
100
100
37
37
D
OCKI_p01
BI-D1870: - - - + + +
Cisplatin: 0 5 10 0 5 10 (μM)
EphA2
EphA2_pS897
EphA2_pY588
β-actin
100
100
100
37
E
Mock
Cisplatin
OCKI_p01
Induction of pEphA2
pS897
pY588
pS897
pY588
Mock
BI-D1870
F
Mock
BI-D1870
OCKI_p01
Cell viability (%)
Cisplatin (μM)
G
CCL-137
LJH685: - - + +
Cisplatin: - + - +
EphA2
EphA2_pS897
EphA2_pY588
β-actin
100
100
100
37
H
Mock
Cisplatin
CCL-137
Induction of pEphA2
pY588
pS897
Mock
LJH685
I
Mock
LJH685
CCL-137
Cell viability (%)
Cisplatin (μM)
J
Mesothelial cell
BI-D1870: - - - + + +
Cisplatin: 0 5 10 0 5 10 (μM)
EphA2
EphA2_pS897
EphA2_pY588
β-actin
100
100
100
37

Appendix Figure S7.

A Representative confocal micrographs show GPRC5A (green) and EphA2 (red), in frozen sections of HGSC patient tumor. S indicates the stroma. Scale bars: 50 µm.
B EphA2 (total and phosphorylated) in OCKI1_p02 and OCKI_p06 cells treated with 0-10 µM cisplatin with and without 10 µM BI-D1870 for 72 h. RSK-EphA2-pS897 inhibition was coupled with EphA2-pY588 upregulation.
C EphA2 (total and phosphorylated) in the GPRC5A^{high} OCKI_p06 HGS cells treated without or with 0-20 µM cisplatin and 0-50 µM LJH685 for 72 h.
D, E EphA2 (D; total and phosphorylated) and corresponding pS897/Y588 ratio (E) in the GPRC5A^{low} OCKI_p01 cells treated with 0-10 µM cisplatin with or without 10 µM BI-D1870 for 72 h. Cisplatin did not prevent EphA2-pS897 and rather decreased EphA2-pY588.
F Cell viability of OCKI_p01 cells treated with 0-20 µM cisplatin with and without 10 µM BI-D1870 for 72 h. Cell viability in these patient-derived HGSC cells with lower GPRC5A was not affected by RSKi. N = 4.
G, H EphA2 (G; total and phosphorylated) and corresponding pS897/Y588 ratio (H) in CCL-137 normal fibroblasts treated with 0-5 µM cisplatin with and without 25 µM LJH685 for 72 h. Upon cisplatin treatment, RSK inhibition did not restore the tumor-suppressive EphA2-pY588.
I Cell viability of CCL-137 normal fibroblasts treated with 0-20 µM cisplatin with and without 25 µM LJH685 for 72 h. Cell viability in these cells was not affected by RSKi. N = 3.
J EphA2 expression and phosphorylation in mesothelial cells treated with 0-10 µM cisplatin and with or without 10 µM BI-D1870 for 72 h.

Data information: In (F, I) are presented as mean (SD). P-values provided in Appendix Table S10, Student’s t-test.

Appendix Table S1. Characteristics of HGSC patients included in the TMA.

**Characteristic**	**Patients (N = 126)**
Median age, years (range)	64 (36-84)
FIGO stage	
I	0 (0%)
II	2 (1.6%)
III	99 (78.6%)
IV	25 (19.8%)
Type of surgery	
Primary	98 (77.8%)
Delayed primary or interval	18 (14.3%)
No surgery	10 (7.9%)
Residual tumor after surgery	
No residual tumor	33 (26.2%)
Residual tumor after surgery	83 (65.9%)
Unknown	10 (7.9%)
First line of chemotherapy	
Platinum (alone or + taxane)	116 (92.1%)
Non-platinum chemotherapy	1 (0.8%)
No chemotherapy	8 (6.3%)
Unknown	1 (0.8%)
Treatment response	
CR	70 (55.6%)
PR	26 (20.6%)
SD	3 (2.4%)
PD	15 (11.9%)
Unknown	12 (9.5%)
Median follow up time, months (range)	36 (1-166)
Survival	
Alive	16 (12.7%)
Dead	110 (87.3%)

Appendix Table S2. Correlation analysis of maximum intensity of GPRC5A staining between primary and metastatic tumor sites. P value was derived from gamma test.

		Staining						P value
		Low		High		Total		
Type	Primary	68	(50.4%)	31	(49.2%)	99	(50%)	0.897
	Metastasis	67	(49.6%)	32	(50.8%)	99	(50%)	
Total		135	(100%)	63	(100%)	198	(100%)	

Appendix Table S3. Correlation analysis between maximum intensity of GPRC5A staining and the clinico-pathological variables. P values were derived from gamma test.

**Clinico-pathological variables**	Primary					Metastatic				
	Staining				P value	Staining				P value
	Low		High			Low		High		
Age at diagnosis (median cutoff, years)					0.507					0.146
≤64	39	52.7%	18	46.2%		33	45.8%	24	60%	
>64	35	47.3%	21	53.8%		39	54.2%	16	40%	
Type of surgery					0.899					0.191
Primary	60	81.1%	32	82.1%		60	83.3%	29	72.5%	
Delayed primary or interval	10	13.5%	5	12.8%		8	11.1%	7	17.5%	
No surgery	4	5.4%	2	5.1%		4	5.6%	4	10%	
Residual tumor after surgery					0.745					0.860
No residual tumor	21	30%	10	27%		20	29.4%	10	27.8%	
Residual tumor	49	70%	27	73%		48	70.6%	26	72.2%	
FIGO stage					**0.006**					0.482
IIC-IIIB	7	9.5%	0	0%		6	8.3%	2	5%	
IIIC-IV	67	90.5%	39	100%		66	91.7%	38	85%	

Appendix Table S4. Survival analysis in primary versus metastatic tumors. P values were derived from Log Rank test.

**OS primary**					
GPRC5A intensity	Total N	Censored N	Mean OS (months)	Std Error	P value
Low	74	12	56.187	5.40	**0.044**
High	39	3	39.236	5.43	
**OS metastatic**					
GPRC5A intensity	Total N	Censored N	Mean OS (months)	Std Error	P value
Low	72	12	59.576	6.02	**0.012**
High	40	3	36.875	5.41	
**PFS primary**					
GPRC5A intensity	Total N	Censored N	Mean PFS (months)	Std Error	P value
Low	66	7	35.248	5.84	0.067
High	35	0	18.314	1.74	
**PFS metastatic**					
GPRC5A intensity	Total N	Censored N	Mean PFS (months)	Std Error	P value
Low	64	7	34.877	5.69	**0.009**
High	36	1	19.833	4.00	

Appendix Table S5. Univariate and multivariate analysis for the association of OS to the clinico-pathological variables and maximum intensity of GPRC5A staining. P values were derived from Cox regression.

**OS Primary**	Univariate				Multivariate			
	N	HR (95% CI)		P value	N	HR (95% CI)		P value
Age at diagnosis (median cutoff, years)	113			**0.002**	107			**0.002**
≤64	57	1	(reference)		55	1	(reference)	
>64	56	1.88	(1.26-2.80)		52	2.03	(1.30-3.15)	
Type of surgery	113				107			
Primary	92	1	(reference)		92	1	(reference)	
Delayed primary or interval	15	1.09	(0.61-1.97)	0.772	15	1.81	(0.94-3.51)	0.078
No surgery	6	3.75	(1.59-8.84)	**0.003**	0	-	-	-
Residual tumor after surgery	107			**0.003**	107			**0.013**
No residual tumor	31	1	(reference)		31	1	(reference)	
Residual tumor	76	2.10	(1.29-3.40)		76	2.09	(1.17-3.74)	
FIGO stage	113			0.122	107			0.626
IIC-IIIB	7	1	(reference)		7	1	(reference)	
IIIC-IV	106	2.04	(0.83-5.02)		100	1.3	(0.45-3.73)	
GPRC5A staining	113			**0.047**	107			**0.032**
Low	74	1	(reference)		70	1	(reference)	
High	39	1.52	(1.01-2.30)		37	1.62	(1.04-2.52)	
**OS Metastatic**		Univariate				Multivariate		
	N	HR (95% CI)		P value	N	HR (95% CI)		P value
Age at diagnosis (median cutoff, years)	112			**0.004**	104			**0.000**
≤64	57	1	(reference)		53	1	(reference)	
>64	55	1.82	(1.22-2.71)		51	2.37	(1.48-3.78)	
Type of surgery	112				104			
Primary	89	1	(reference)		89	1	(reference)	
Delayed primary or interval	15	1.06	(0.59-1.91)	0.846	15	1.42	(0.76-2.66)	0.275
No surgery	8	4.94	(2.29-10.66)	**0.000**	-	-	-	-
Residual tumor after surgery	104			**0.002**	104			**0.029**
No residual tumor	30	1	(reference)		30	1	(reference)	
Residual tumor	74	2.16	(1.33-3.52)		74	1.93	(1.07-3.47)	
FIGO stage	112			0.128	104			0.312
IIC-IIIB	8	1	(reference)		8	1	(reference)	
IIIC-IV	104	0.526	(0.23-1.20)		96	1.68	(0.61-4.59)	
GPRC5A staining	112			**0.013**	104			**0.002**
Low	72	1	(reference)		68	1	(reference)	
High	40	1.69	(1.12-2.57)		36	2.12	(1.33-3.37)	

Appendix Table S6. Univariate and multivariate analysis for the association of PFS to the clinico-pathological variables and maximum intensity of GPRC5A staining. P values were derived from Cox regression.

**PFS Primary**	Univariate				Multivariate			
	N	HR (95% CI)		P value	N	HR (95% CI)		P value
Age at diagnosis (median cutoff, years)	101			0.630	97			0.719
≤64	53	1	(reference)		51	1	(reference)	
>64	48	1.11	(0.74-1.66)		46	1.09	(0.69-1.72)	
Type of surgery	101				97			
Primary	82	1	(reference)		82	1	(reference)	
Delayed primary or interval	15	0.97	(0.54-1.76)	0.931	15	1.20	(0.61-2.34)	0.598
No surgery	4	1.31	(0.47-3.59)	0.606	-	-	-	-
Residual tumor after surgery	97			**0.018**	97			0.092
No residual tumor	30	1	(reference)		30	1	(reference)	
Residual tumor	67	1.76	(1.10-2.80)		67	1.63	(0.92-2.88)	
FIGO stage	101			0.060	97			0.380
IIC-IIIB	6	1	(reference)		6	1	(reference)	
IIIC-IV	95	2.64	(0.96-7.23)		91	1.70	(0.52-5.52)	
GPRC5A staining	101			0.077	97			0.143
Low	66	1	(reference)		64	1	(reference)	
High	35	1.47	(0.96-2.26)		33	1.40	(0.89-2.19)	
**PFS Metastatic**		Univariate				Multivariate		
	N	HR (95% CI)		P value	N	HR (95% CI)		P value
Age at diagnosis (median cutoff, years)	100			0.559	94			0.207
≤64	53	1	(reference)		49	1	(reference)	
>64	47	1.13	(0.75-1.70)		45	1.35	(0.85-2.15)	
Type of surgery	100				94			
Primary	79	1	(reference)		79	1	(reference)	
Delayed primary or interval	15	0.96	(0.52-1.77)	0.889	15	1.18	(0.60-2.30)	0.638
No surgery	6	2.10	(0.90-4.89)	0.084	-	-	-	-
Residual tumor after surgery	94			**0.010**	94			0.131
No residual tumor	29	1	(reference)		29	1	(reference)	
Residual tumor	65	1.80	(1.16-3.05)		65	1.57	(0.87-2.81)	
FIGO stage	100			0.055	94			0.237
IIC-IIIB	7	1	(reference)		7	1	(reference)	
IIIC-IV	93	2.43	(0.98-6.02)		87	1.92	(0.65-5.68)	
GPRC5A staining	100			**0.012**	94			**0.043**
Low	64	1	(reference)		62	1	(reference)	
High	36	1.72	(1.13-2.63)		32	1.59	(1.01-2.49)	

Appendix Table S7. Correlation of ORR and treatment sensitivity with maximum intensity of GPRC5A staining. P values were derived from Pearson Chi-square test.

**PRIMARY**	GPRC5A staining							
		Low		High		Total		P value
**ORR**	Responsive	61	(89.7%)	25	(73.5%)	86	(84.3%)	**0.034**
	Non-responsive	7	(10.3%)	9	(26.5%)	16	(15.7%)	
Total		68	(100%)	34	(100%)	102	(100%)	
		Low		High		Total		P value
**Treatment sensitivity**	Sensitive	60	(90.9%)	25	(71.4%)	85	(84.2%)	**0.011**
	Resistant	6	(9.1%)	10	(28.6%)	16	(15.8%)	
Total		66	(100%)	35	(100%)	101	(100%)	
**METASTATIC**	GPRC5A staining							
		Low		High		Total		P value
**ORR**	Responsive	56	(84.8%)	30	(83.3%)	86	(84.3%)	0.841
	Non-responsive	10	(15.2%)	6	(16.7%)	16	(15.7%)	
Total		66	(100%)	36	(100%)	102	(100%)	
		Low		High		Total		P value
**Treatment sensitivity**	Sensitive	55	(85.9%)	31	(81.6%)	86	(84.3%)	0.558
	Resistant	9	(14.1%)	7	(18.4%)	16	(15.7%)	
Total		64	(100%)	38	(100%)	102	(100%)	

Appendix Table S8. Probe IDs from The Cancer Cell Line Encyclopedia (https://portals.broadinstitute.org/ccle) for the data used in this study.

**Description**	**Probe ID**				
**CDH1**	201130_s_at	201131_s_at			
**CDH2**	203440_at	203441_s_at			
**VIM**	1555938_x_at	201426_s_at			
**PAX8**	121_at	207921_x_at	207923_x_at	207924_x_at	209552_at
	213917_at	214528_s_at	221990_at		
**KRT7**	1558393_at	1558394_s_at	209016_s_at	214031_s_at	
**CD44**	1557905_s_at	1565868_at	204489_s_at	204490_s_at	209835_x_at
	210916_s_at	212014_x_at	212063_at	216056_at	217523_at
	229221_at	234411_x_at	234418_x_at		
**EPHA2**	203499_at				
**EFNA1**	202023_at				
**RSK1 (RPS6KA1)**	203379_at				
**RSK2 (RPS6KA3)**	203843_at	226335_at			
**RSK3 (RPS6KA2)**	1557970_s_at	204906_at	212912_at		
**RSK4 (RPS6KA4)**	204632_at	230544_at			

Appendix Table S9. siRNA sequences

**siRNA**	**Target sequence**
GPRC5A#2	5'-GAGGCTAAAGATCACCCTAAA-3'
GPRC5A#5	5'-CAACTCAAGTTTAGACCCTTA-3'

Appendix Table S10. Exact significant p-values in figures.

Figure	Comparison	p-value	Statistical test
Fig 1C	OCKI_p01 cis/mock	0.006	Two-sided Student t-test
Fig 1C	OCKI_p03 cis/mock	0.008	Two-sided Student t-test
Fig 1C	OCKI_p06 cis/mock	0.007	Two-sided Student t-test
Fig 2B	OVCAR3 pS897/pY588 (0-5 µM cis)	0.047	Two-sided Student t-test
Fig 2B	OVCAR3 pS897/pY588 (0-10 µM cis)	0.048	Two-sided Student t-test
Fig 2B	OVCAR4 pS897/pY588 (0-5 µM cis)	0.012	Two-sided Student t-test
Fig 2B	OVCAR4 pS897/pY588 (0-10 µM cis)	0.033	Two-sided Student t-test
Fig 2B	OVCAR4 pS897/pY588 (0-20 µM cis)	0.016	Two-sided Student t-test
Fig 2B	OVCAR8 pS897/pY588 (0-5 µM cis)	0.019	Two-sided Student t-test
Fig 2B	OVCAR8 pS897/pY588 (0-10 µM cis)	0.012	Two-sided Student t-test
Fig 2B	OVCAR8 pS897/pY588 (0-20 µM cis)	0.014	Two-sided Student t-test
Fig 2B	OVCAR3-OVCAR8 pS897/pY588 (0 µM cis)	0.023	Two-sided Student t-test
Fig 2B	OVCAR4-OVCAR8 pS897/pY588 (0 µM cis)	0.011	Two-sided Student t-test
Fig 2D	OVCAR8-OVCAR3 viability (20 µM cis)	<0.0001	Two-sided Student t-test
Fig 2D	OVCAR8-OVCAR4 viability (20 µM cis)	0.0006	Two-sided Student t-test
Fig 2E	HGSC pS897/pY588 (0-5 µM cis)	0.004	Two-sided Student t-test
Fig 2E	HGSC pS897/pY588 (0-10 µM cis)	0.011	Two-sided Student t-test
Fig 2E	HGSC pS897/pY588 (0-20 µM cis)	0.02	Two-sided Student t-test
Fig 2H	OVCAR4 pS897/pY588 (0-80 µM carbo)	0.014	Two-sided Student t-test
Fig 2H	OVCAR4-OVCAR8 pS897/pY588 (0 µM carbo)	0.048	Two-sided Student t-test
Fig 3B	Mock – carboplatin tumor size (day 43)	0.016	Mann-Whitney U test
Fig 3B	Mock – carboplatin tumor size (day 47)	0.008	Mann-Whitney U test
Fig 3B	Mock – carboplatin tumor size (day 50)	0.009	Mann-Whitney U test
Fig 3B	Mock – carboplatin tumor size (day 53)	0.008	Mann-Whitney U test
Fig 3C	Mock – carboplatin ascites volume (day 53)	0.006	Mann-Whitney U test

Fig 3F	Mock – carboplatin EphA2	0.007	Mann-Whitney U test
Fig 3G	Mock – carboplatin EphA2_pS897	0.009	Mann-Whitney U test
Fig 3I	Mock – carboplatin clCasp3	0.012	Mann-Whitney U test
Fig 4B	TYK-nu pS897/pY588 (0-5 µM cis)	0.004	Two-sided Student t-test
Fig 4B	TYK-nu pS897/pY588 (0-10 µM cis)	0.009	Two-sided Student t-test
Fig 4B	TYK-nu.R pS897/pY588 (0-5 µM cis)	0.033	Two-sided Student t-test
Fig 4B	TYK-nu.R pS897/pY588 (0-10 µM cis)	0.014	Two-sided Student t-test
Fig 4B	TYK-nu - TYK-nu.R pS897/pY588 (0 µM cis)	0.004	Two-sided Student t-test
Fig 4F	TYK-nu pS897/GAPDH (mock-BI-1870)	0.003	Two-sided Student t-test
Fig 5A	OVCAR4 viability mock-BI-D1870 (5 µM cis)	<0.0001	Two-sided Student t-test
Fig 5A	OVCAR4 viability mock-BI-D1870 (10 µM cis)	0.001	Two-sided Student t-test
Fig 5A	OVCAR8 viability mock-BI-D1870 (20 µM cis)	0.001	Two-sided Student t-test
Fig 5A	TYK-nu viability mock-BI-D1870 (0 µM cis)	0.025	Two-sided Student t-test
Fig 5A	TYK-nu.R viability mock-BI-D1870 (0 µM cis)	0.002	Two-sided Student t-test
Fig 5A	TYK-nu.R viability mock-BI-D1870 (5 µM cis)	<0.0001	Two-sided Student t-test
Fig 5A	TYK-nu.R viability mock-BI-D1870 (10 µM cis)	0.004	Two-sided Student t-test
Fig 5B	OVCAR8 viability mock-LJH685 (10 µM cis)	0.041	Two-sided Student t-test
Fig 5B	OVCAR8 viability mock-LJH685 (20 µM cis)	0.011	Two-sided Student t-test
Fig 6B	OVCAR8 GPRC5A 46/41 kDa (cis)	0.011	Two-sided Student t-test
Fig 6B	OVCAR8 GPRC5A 46/41 kDa (LJH685)	0.025	Two-sided Student t-test
Fig 6B	TYK-nu.R GPRC5A 46/41 kDa (cis)	0.011	Two-sided Student t-test
Fig 6B	TYK-nu.R GPRC5A 46/41 kDa (LJH685)	0.004	Two-sided Student t-test
Fig 6F	TYK-nu.R viability siNT-siRSK1	0.016	Two-sided Student t-test
Fig 6F	TYK-nu.R viability siNT-siRSK2	0.006	Two-sided Student t-test
Fig 6F	TYK-nu.R viability siNT-siRSK1+2	0.007	Two-sided Student t-test
Fig 7B	clCasp3+ OVCAR8 (mock-cis)	0.029	Two-sided Student t-test
Fig 7B	clCasp3+ OVCAR8 (mock-combinatorial)	0.002	Two-sided Student t-test

Fig 7B	clCasp3+ co-culture (mock-combinatorial)	0.022	Two-sided Student t-test
Fig 7D	clCasp3+ co-culture (mock-cis)	0.023	Two-sided Student t-test
Fig 7D	clCasp3+ co-culture (cis-combinatorial)	0.002	Two-sided Student t-test
Fig 7D	clCasp3+ co-culture (mock-combinatorial)	0.003	Two-sided Student t-test
Fig 7F	Mock – carboplatin+BI-1870 TUNEL+	0.0143	Mann-Whitney U test
Fig 8E	OCKI_p02 viability mock-BI-D1870 (10 μM cis)	0.027	Two-sided Student t-test
Fig 8E	OCKI_p02 viability mock-BI-D1870 (20 μM cis)	0.02	Two-sided Student t-test
Fig 8E	OCKI_p06 viability mock-BI-D1870 (20 μM cis)	0.012	Two-sided Student t-test
Fig EV1E	clCasp3+ OCKI_p13 (mock-cis)	0.022	Two-sided Student t-test
Fig EV3C	OVCAR4 viability mock-RSK1/2 (5 μM cis)	0.003 / 0.048	Two-sided Student t-test
Fig EV3C	OVCAR4 viability mock-RSK1 (10 μM cis)	0.0005	Two-sided Student t-test
Fig EV3D	OVCAR8 viability mock-RSK1 (20 μM cis)	0.049	Two-sided Student t-test
Fig EV3E	OVCAR4 viability mock-GPRC5A (2.5 μM cis)	0.022	Two-sided Student t-test
Fig EV3E	OVCAR4 viability mock-GPRC5A (10 μM cis)	0.002	Two-sided Student t-test
Fig EV3F	OVCAR4 viability mock-EphA2 (5 μM cis)	0.029	Two-sided Student t-test
Fig EV4B	GPRC5A-EphA2 co-localization (mock-carbo)	0.0002	Mann-Whitney U test
Fig S2C	OCKI_p06 Tp53 intensity (nutlin/mock)	0.011	Two-sided Student t-test
Fig S2C	ANR8 Tp53 intensity (nutlin/mock)	0.01	Two-sided Student t-test
Fig S3B	OVCAR8 viability 0-100 μM LJH685	0.039	Two-sided Student t-test
Fig S3B	TYK-nu viability 0-100 μM LJH685	0.012	Two-sided Student t-test
Fig S3E	OVCAR4 viability mock-Tram. 0.5 μM / 1 μM (0 μM cis)	Both <0.0001	Two-sided Student t-test
Fig S3E	OVCAR4 viability mock-Tram. 0.5 μM / 1 μM (2.5 μM cis)	Both <0.0001	Two-sided Student t-test
Fig S3E	OVCAR4 viability mock-Tram. 0.5 μM / 1 μM (5 μM cis)	0.0003 / 0.0004	Two-sided Student t-test
Fig S3H	TUNEL+ tumor (mock-carbo)	0.049	Mann-Whitney U test
Fig S3H	TUNEL+ tumor (mock-combinatorial)	0.021	Mann-Whitney U test
