# Supplementary material for: Adaptive RSK‐EphA2‐GPRC5A signaling switch triggers chemotherapy resistance in ovarian cancer
Source: EMBO Mol Med. 2020 Mar 2;12(4):e11177. doi: 10.15252/emmm.201911177 (PMC7136956; doi:10.15252/emmm.201911177)
Supplement: Supplementary file 2 — Expanded View Figures PDF [file EMMM-12-e11177-s002.pdf]

Expanded View Figures

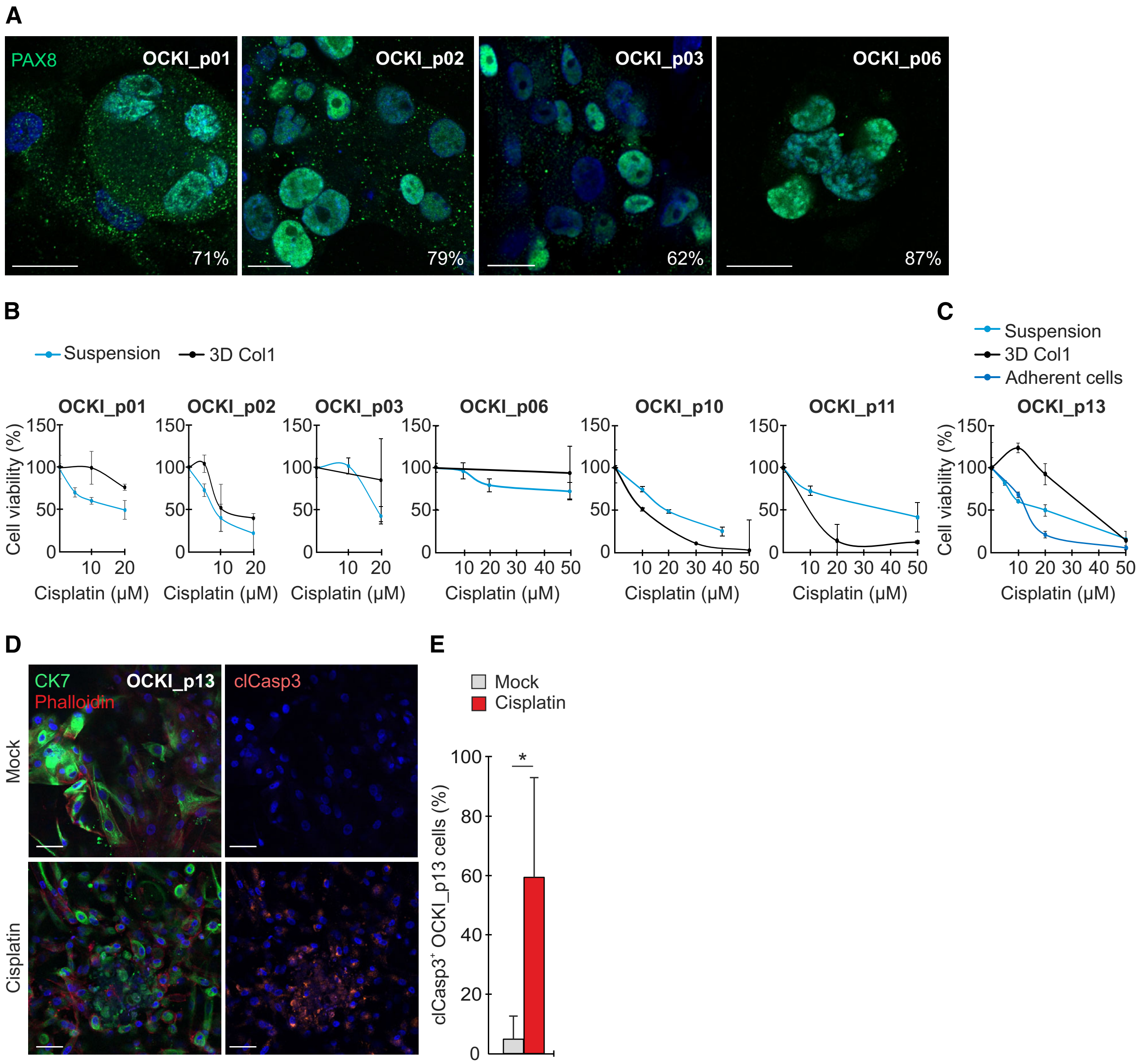

Figure EV1. Patient-derived HGSC cells show variable cisplatin resistance in 3D collagen.

A Confocal micrographs show PAX8 (green) in primary patient-derived HGSC cells after 7-days culture in 3D collagen. Cells were 62–87% positive for the nuclear HGSC marker. Scale bars: 20 μm.

B, C Viability of patient-derived HGSC cells cultured for 7 days in ascites-like suspension or 3D collagen (B, C; viability of freshly isolated primary cells could only be assessed once with $N = 3$ experimental replicates) as well as in an adherent culture (C; $N = 3$) after 0–50 μM cisplatin treatment for 72 h. Cells derived from patient OCKI_p01, OCKI_p03, OCKI_p06, and OCKI_p13 showed treatment resistance particularly when embedded in 3D collagen.

D, E Representative confocal micrographs show cytokeratin 7 (CK7, green), phalloidin (F-actin, red), and cleaved caspase-3 (clCasp3, orange) in 3D OCKI_p13 HGSC culture treated without or with 20 μM cisplatin for 72 h, 4 days after 3D embedding. Corresponding quantification for clCasp3 (E). Scale bars: 50 μm. $N = 4$.

Data information: In (B, C and E), data are presented as mean (SD). $^*P < 0.05$. Exact P-values are provided in Appendix Table S10, Student's t-test.

© 2020 The Authors

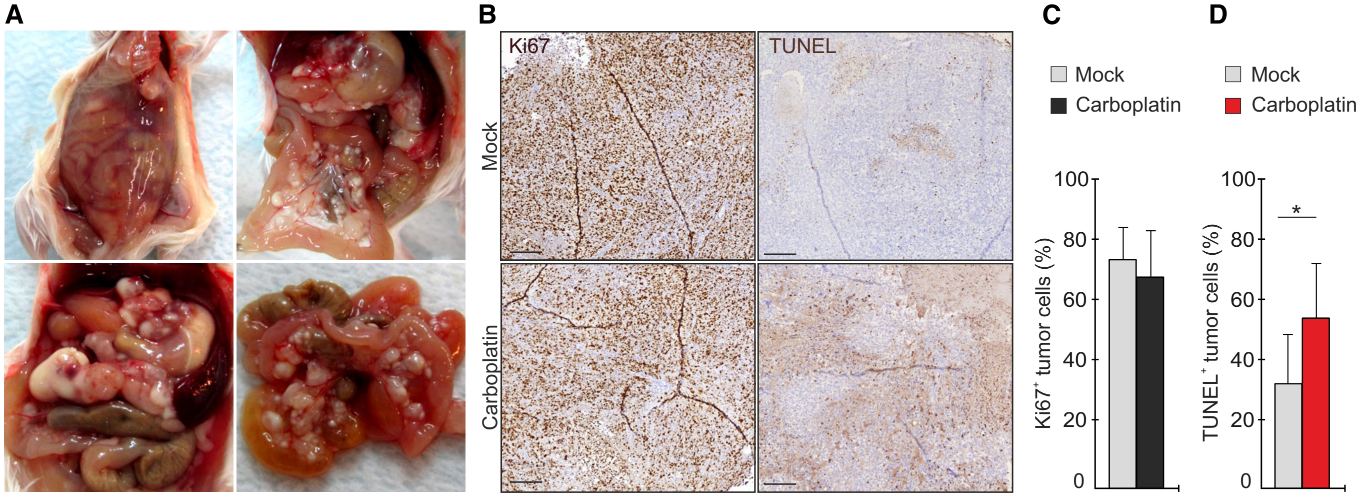

Figure EV2. OVCAR xenograft tumor model mimics human HGSC.

A Representative images of tumors in OVCAR4 xenograft mice, mimicking the development of HGSC in patients: accumulation of ascites in the peritoneal cavity, widely disseminated metastatic foci, and multiple nodules located in the omentum and peritoneal membrane.

B–D Histological assessment of proliferation by Ki67 and apoptosis by TUNEL (B) and corresponding quantification of Ki67$^+$ (C) and TUNEL$^+$ (D) OVCAR4 in xenograft tumors of mock and carboplatin-treated mice. *N* = 5 mice/group. Scale: 200 μm. Carboplatin treatment significantly induced apoptosis but had no major effect on proliferation.

Data information: In (C, D), data are presented as mean (SD). $^*P < 0.05$. Exact *P*-values are provided in Appendix Table S10, Mann–Whitney *U*-test.

 © 2020 The Authors

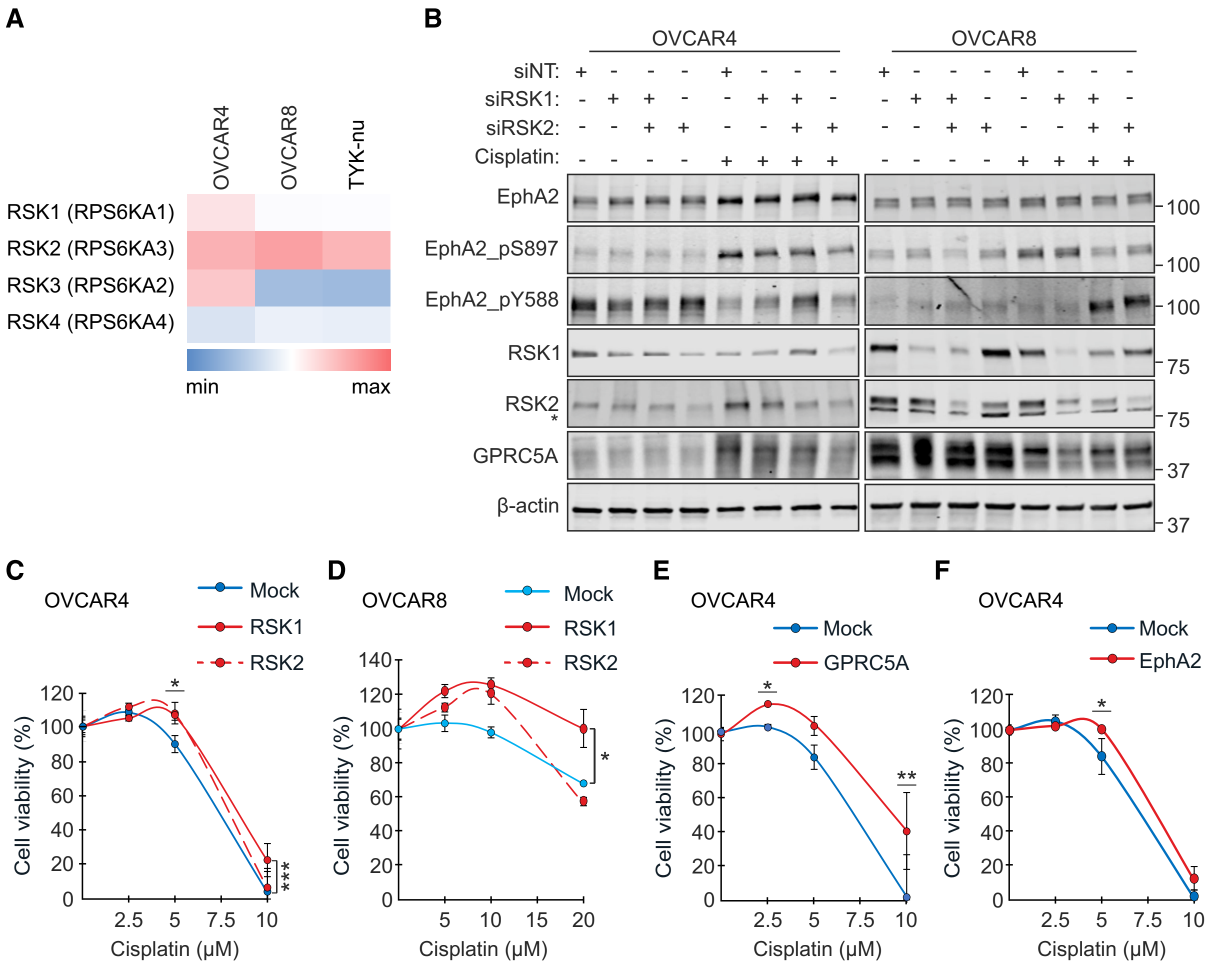

Figure EV3. RSK1/2 specifically regulates EphA2-GPRC5A signaling axis.

A Heat map for RSK1 (RPS6KA1), RSK2 (RPS6KA3), RSK3 (RPS6KA2), and RSK4 (RPS6KA4) mRNAs in OVCAR4, OVCAR8, and TYK-nu. The cells expressed most prominently RSK1/2 over RSK3/RSK4. Data obtained from the Cancer Cell Line Encyclopedia (https://portals.broadinstitute.org/ccle) and illustrated per cell line. See Appendix Table S8 for probe IDs.

B EphA2, RSK1, and RSK2 protein levels and EphA2 phosphorylation as indicated in RSK1/2-depleted and cisplatin-treated OC cells 5 days after transfection. OVCAR4 was treated with 0–5 μM and OVCAR8 with 0–10 μM cisplatin for 72 h. Asterisk indicates unspecific band.

C, D Cell viability of RSK1 and RSK2 overexpressing OVCAR4 (C; *N* = 4) and OVCAR8 (D; *N* = 3) after treatment with 0–20 μM cisplatin for 72 h.

E, F Viability of OVCAR4 overexpressing GPRC5A (E; *N* = 4) or EphA2 (F; *N* = 3) after treatment with 0–10 μM cisplatin for 72 h.

Data information: In (C–F), data are presented as mean (SD). *$P < 0.05$, **$P < 0.01$, ***$P < 0.001$. Exact *P*-values are provided in Appendix Table S10, Student's *t*-test. Source data are available online for this figure.

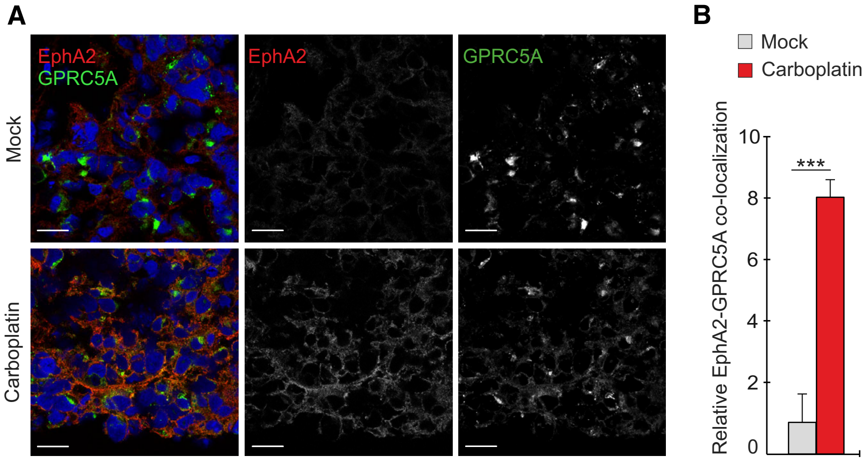

Figure EV4. Co-localization of GPRC5A and EphA2 is induced upon platinum treatment *in vivo*.

A, B Representative confocal micrographs (A) of EphA2 (red) and GPRC5A (green) and corresponding co-localization quantification (B) in OVCAR4 xenograft tumors of mock and carboplatin-treated mice. $N = 5$ mice/group. Scale bars: 20 µm.

Data information: In (B), data are presented as mean (SD). ***$P < 0.001$. Exact *P*-values are provided in Appendix Table S10, Mann–Whitney *U*-test.

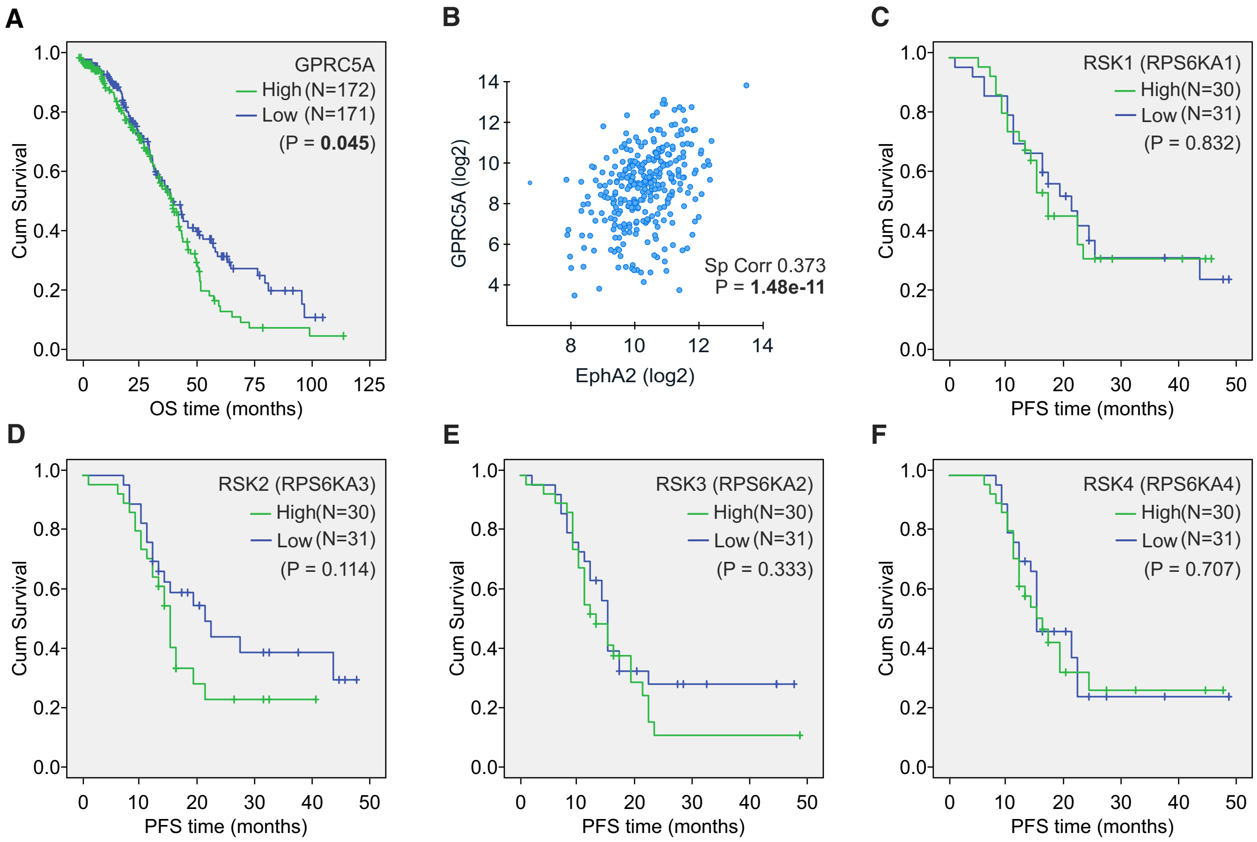

Figure EV5. GPRC5A is co-expressed with EphA2 and associates with worse survival of OC patients.

A Kaplan–Meier survival curve illustrates the overall survival (OS) of patients with high or low (top 40% vs. bottom 40%) GPRC5A mRNA expression. Mean OS was 46 months for $GPRC5A^{high}$ vs. 56 months for $GPRC5A^{low}$.

B Scatter plot illustrates the correlation of EphA2 and GPRC5A mRNA in OC (using TCGA by cBioPortal for Cancer Genomics).

C–F Kaplan–Meier survival curves illustrate the progression-free survival (PFS) of patients with high or low (top 25% vs. bottom 25%) RSK1 (RPS6KA1; C), RSK2 (RPS6KA3; D), RSK3 (RPS6KA2; E), and RSK4 (RPS6KA4; F) mRNA expression. No significant differences in PFS were observed in these survival analyses.

Data information: In (A, C–F), logrank test was used. In (B), Spearman's rank test was used.

 © 2020 The Authors
