# Supplementary figures and images for: Adaptive RSK‐EphA2‐GPRC5A signaling switch triggers chemotherapy resistance in ovarian cancer
Source: EMBO Mol Med. 2020 Mar 2;12(4):e11177. doi: 10.15252/emmm.201911177 (PMC7136956; doi:10.15252/emmm.201911177)

**B**

OVCAR4

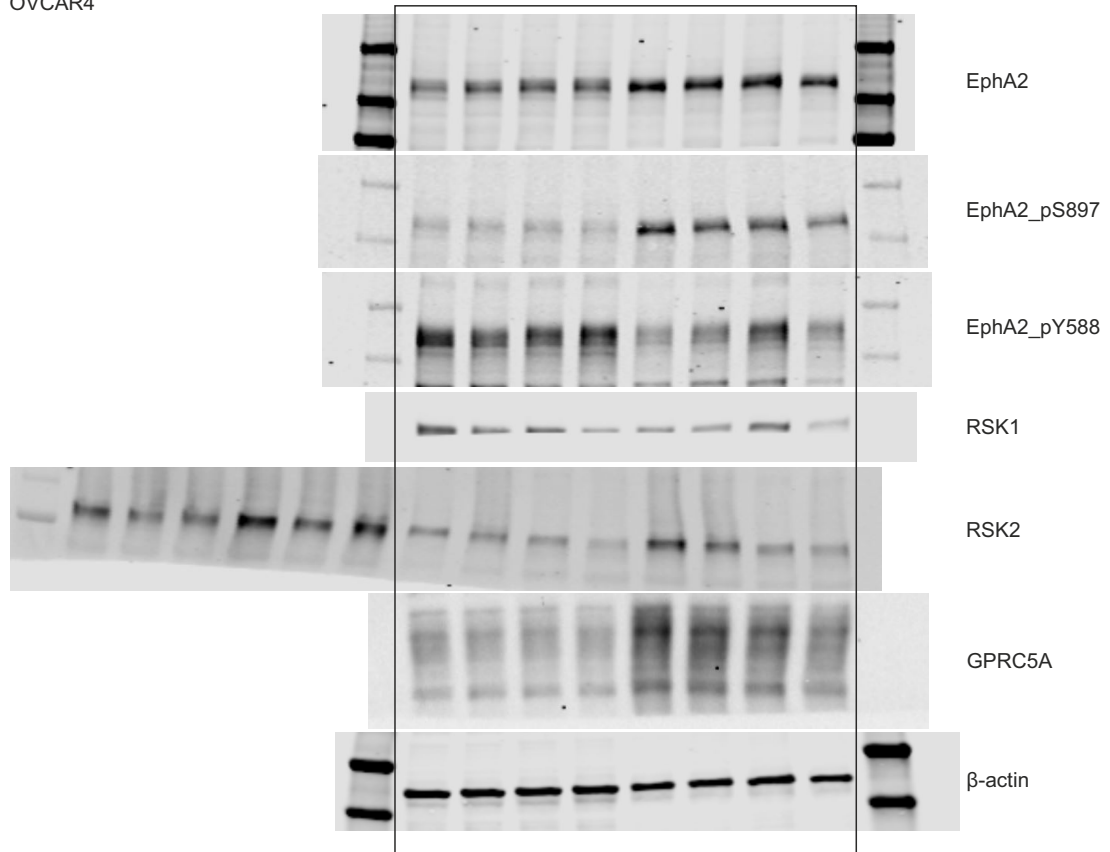

OVCAR8

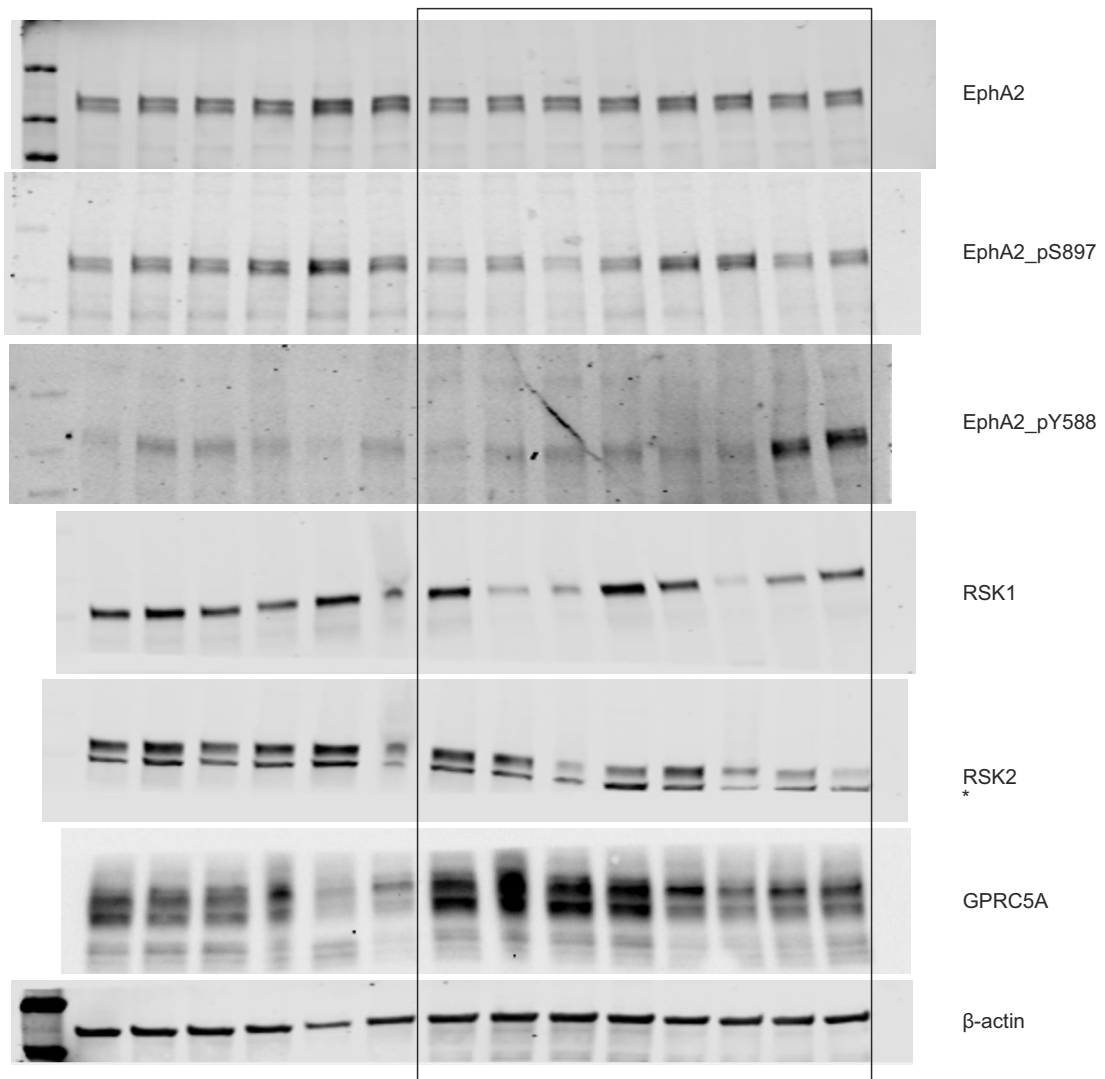

Supplement: Supplementary file 3 — Source Data for Expanded View [file EMMM-12-e11177-s009.zip › Source_data_Fig_EV3.pdf]

**A**

OVCAR3

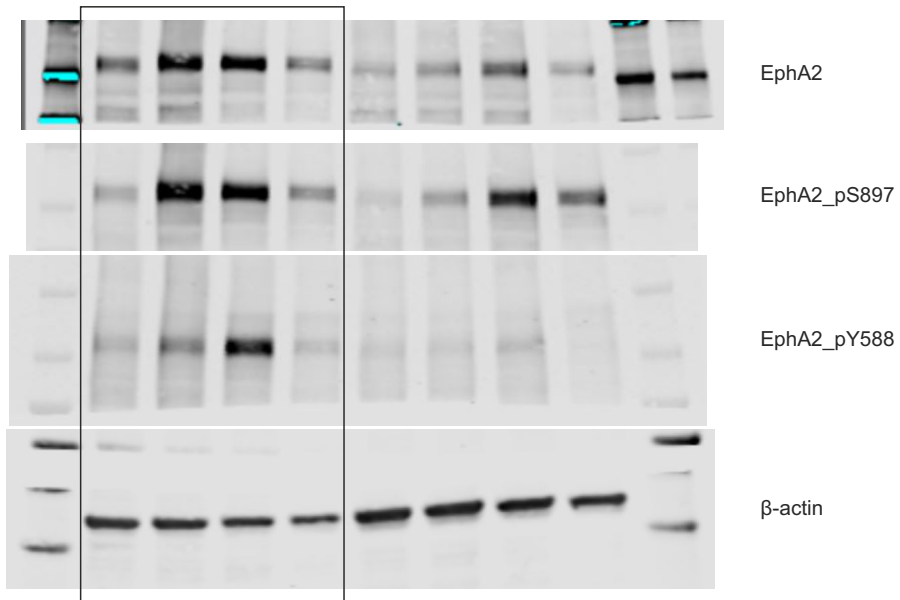

OVCAR4

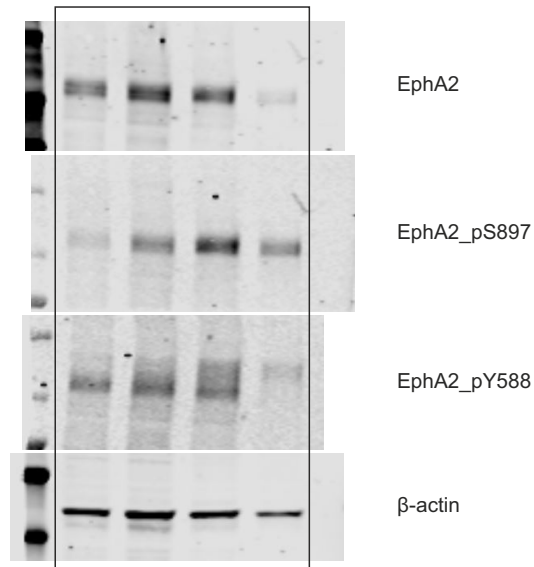

OVCAR8

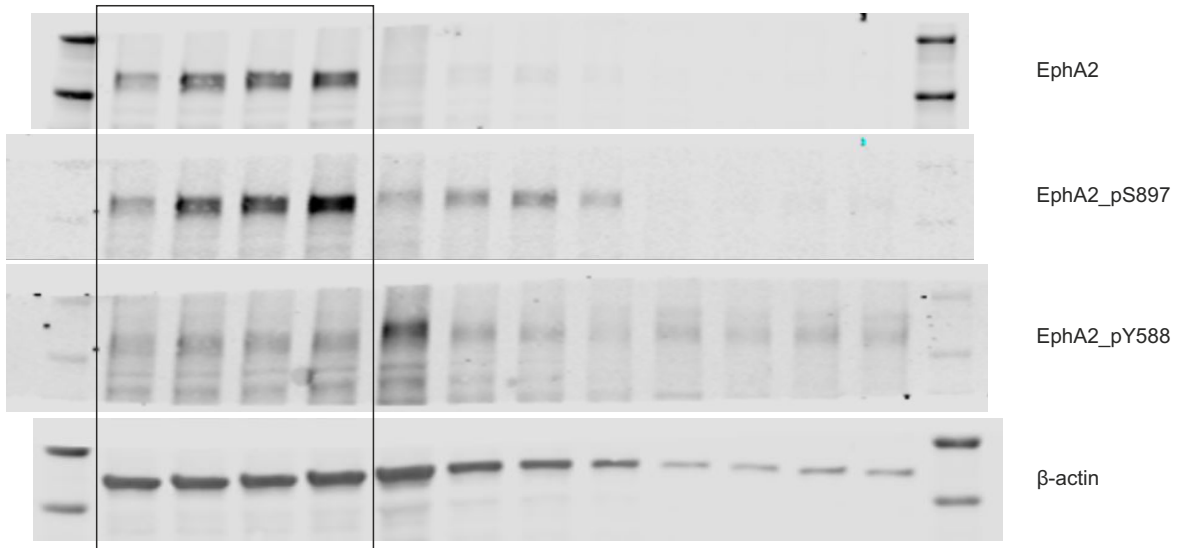

**C**

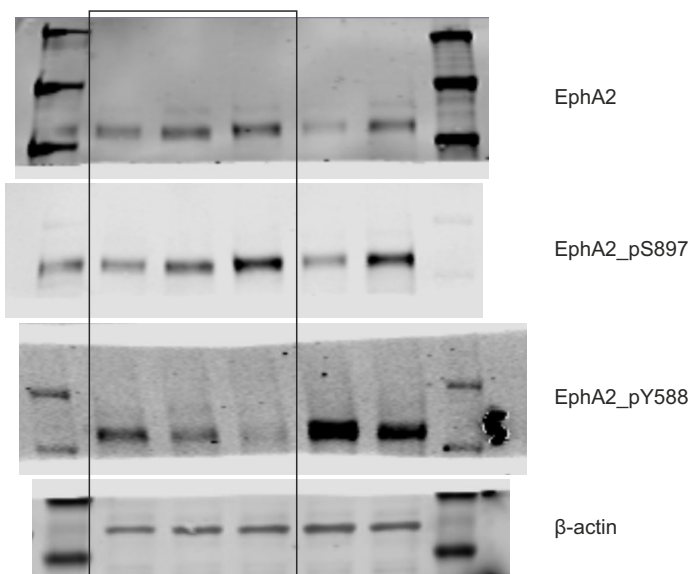

**G**

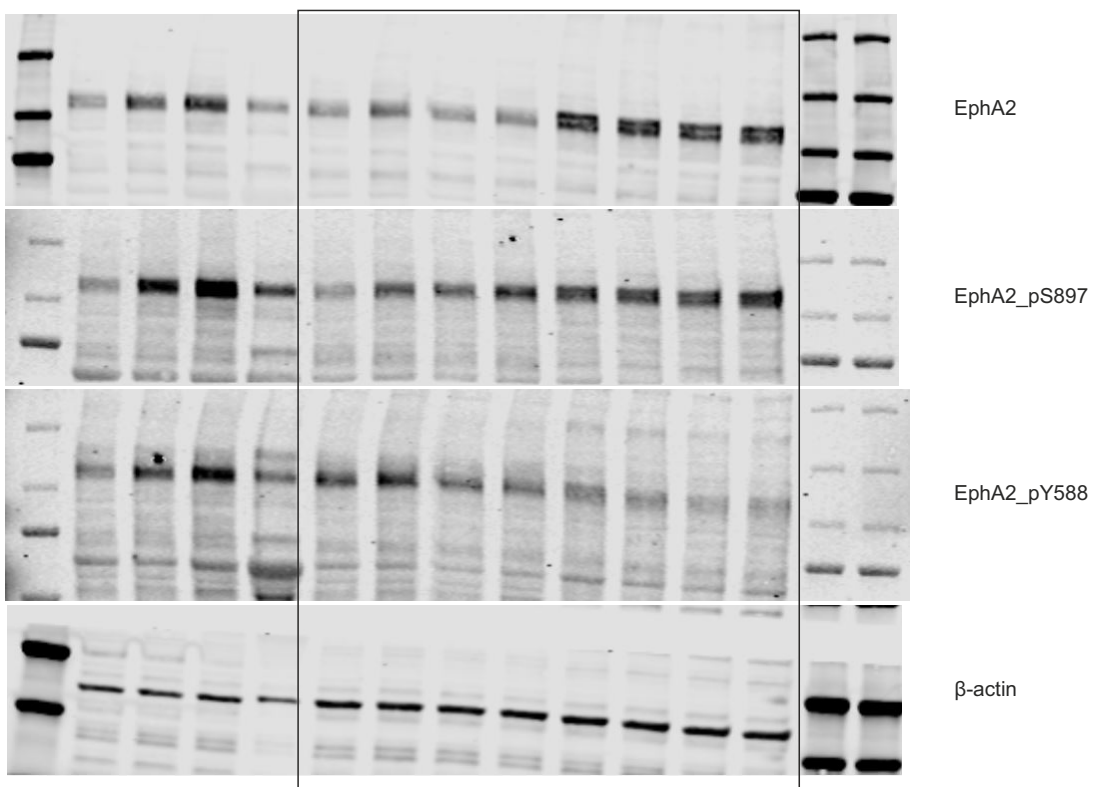

Supplement: Supplementary file 5 — Source Data for Figure 2 [file EMMM-12-e11177-s003.pdf]

**A**

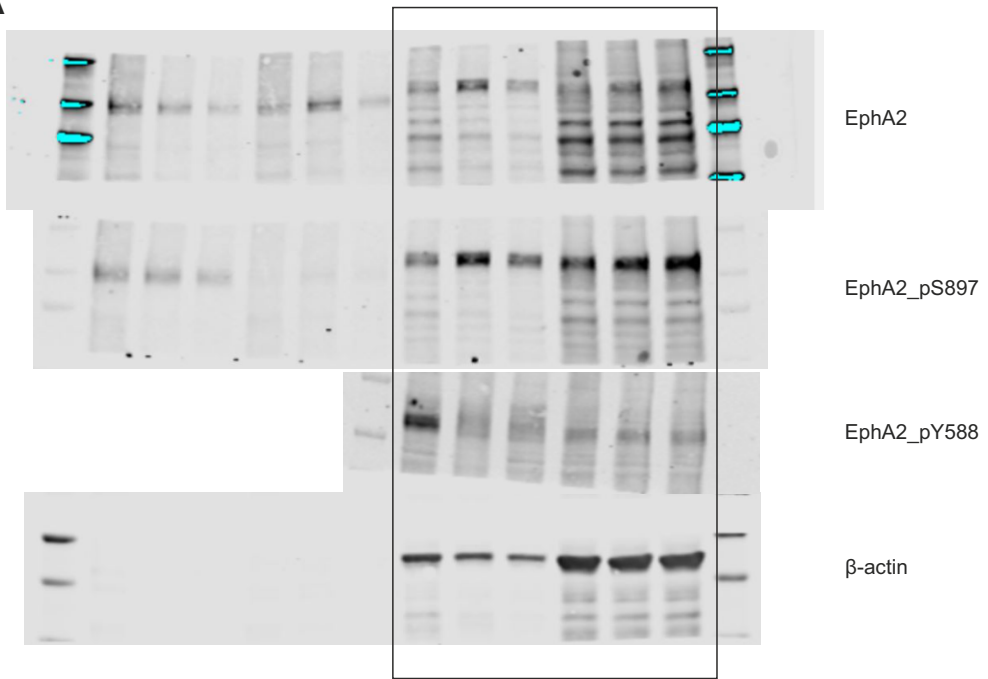

**C**

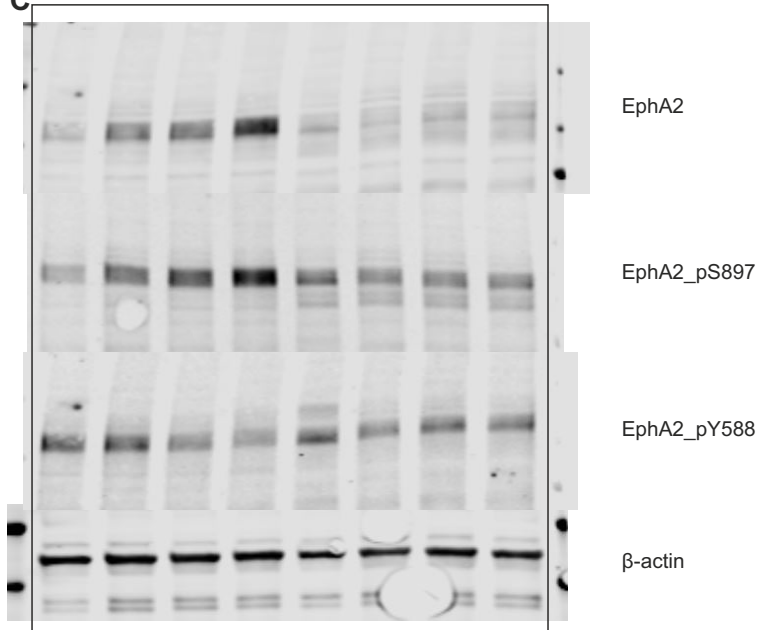

**D**

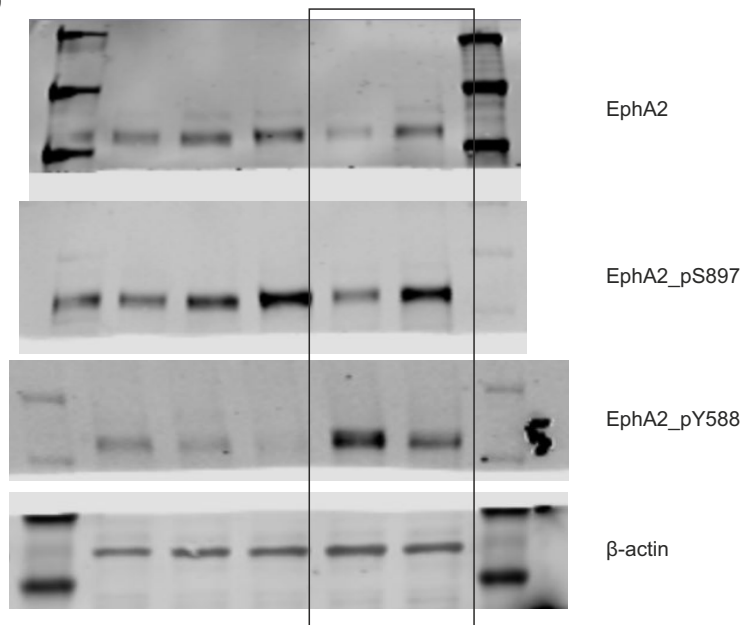

**E**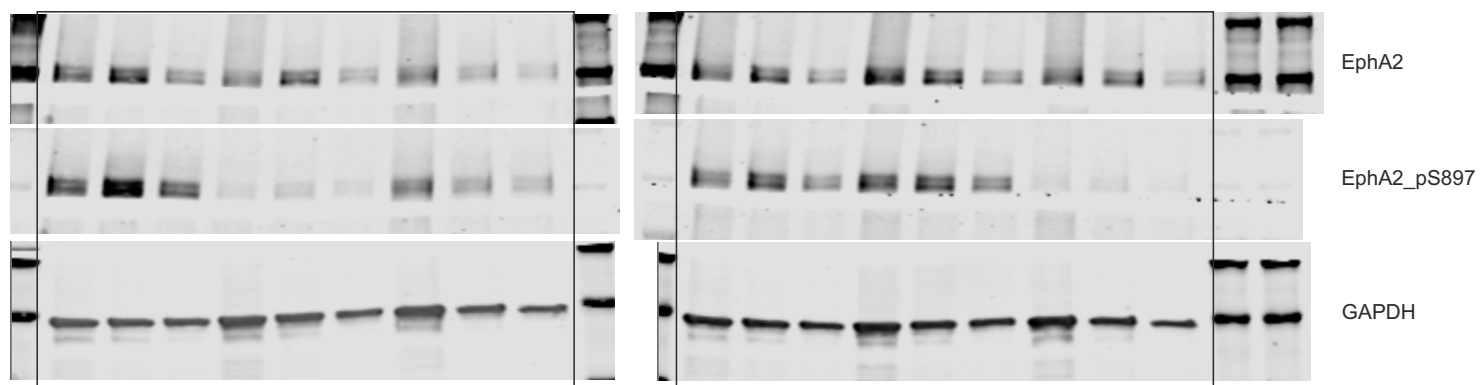**G**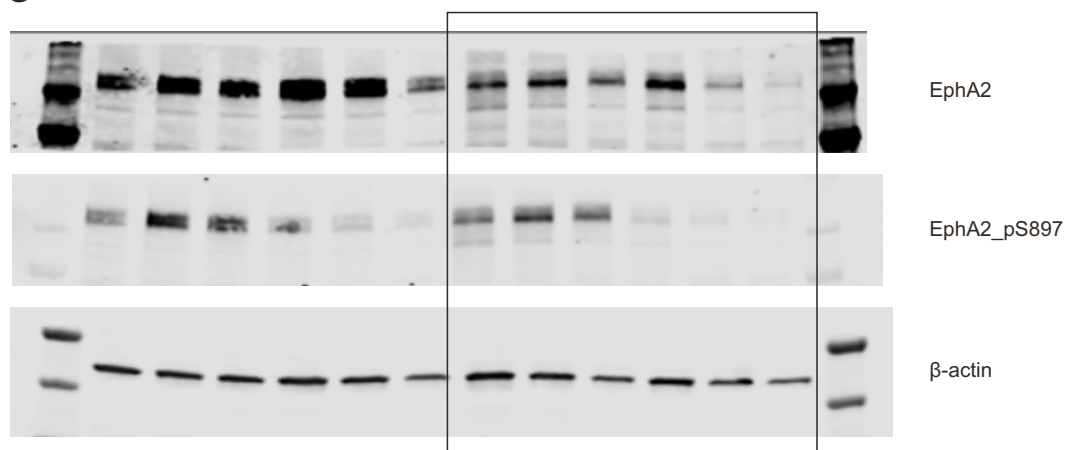**H-I**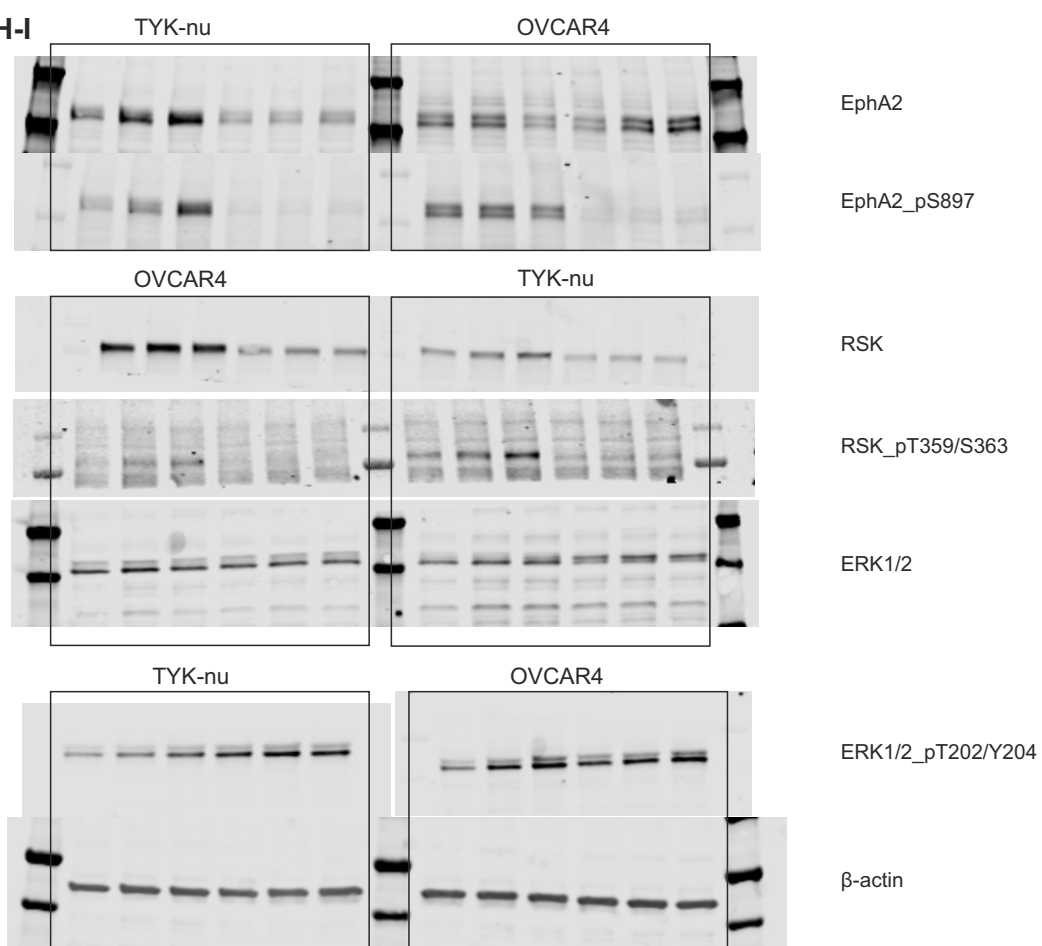

Supplement: Supplementary file 7 — Source Data for Figure 4 [file EMMM-12-e11177-s005.pdf]

**C**

OVCA4

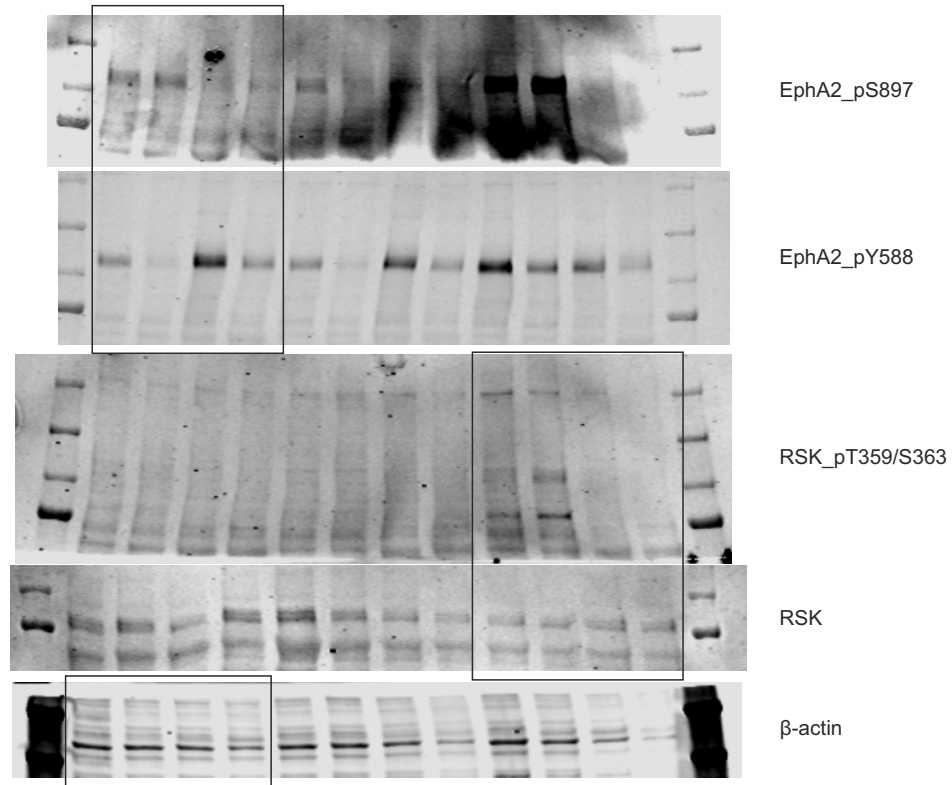

OVCA8

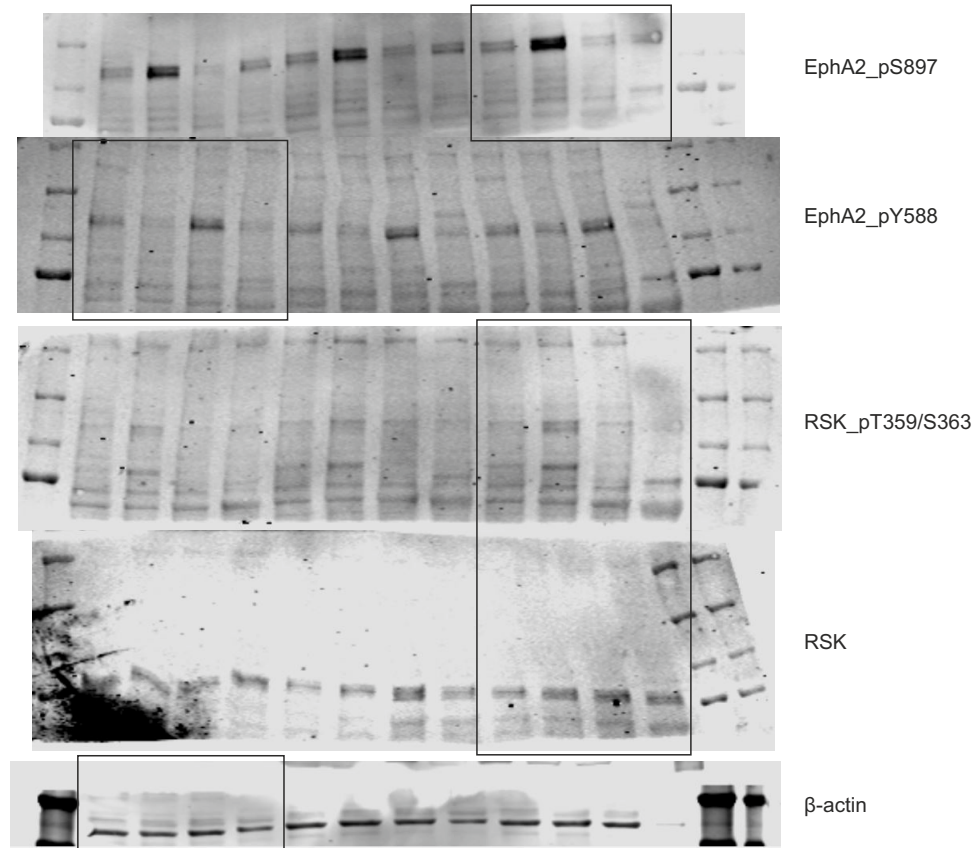

### C (continued)

TYK-nu

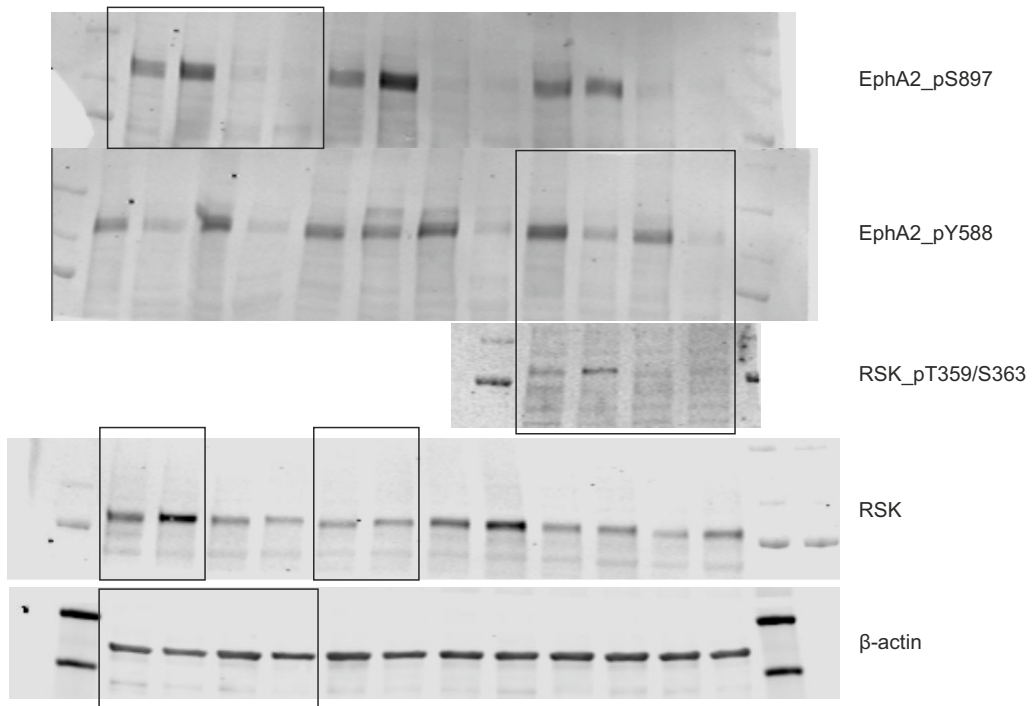

TYK-nu.R

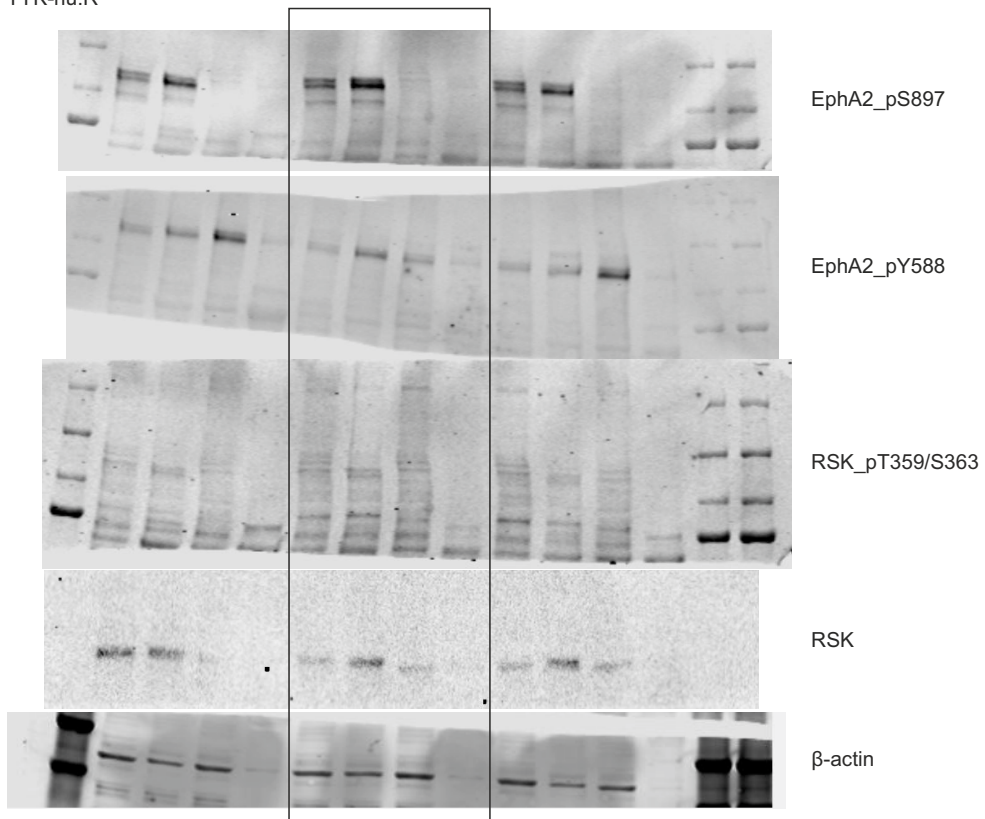

Supplement: Supplementary file 8 — Source Data for Figure 5 [file EMMM-12-e11177-s006.pdf]

**A**

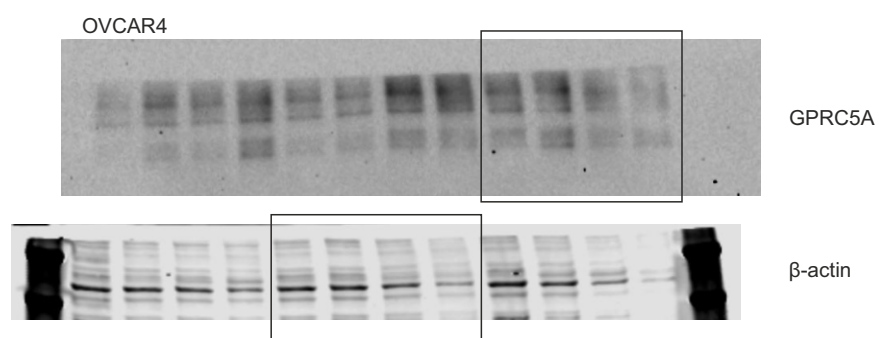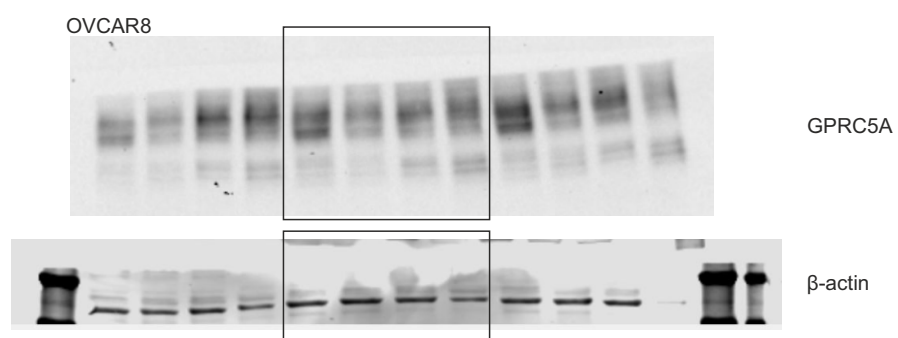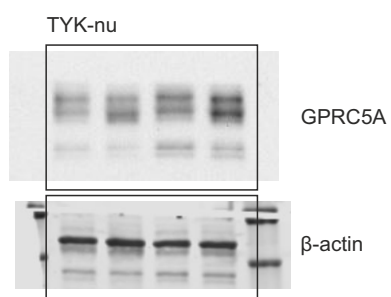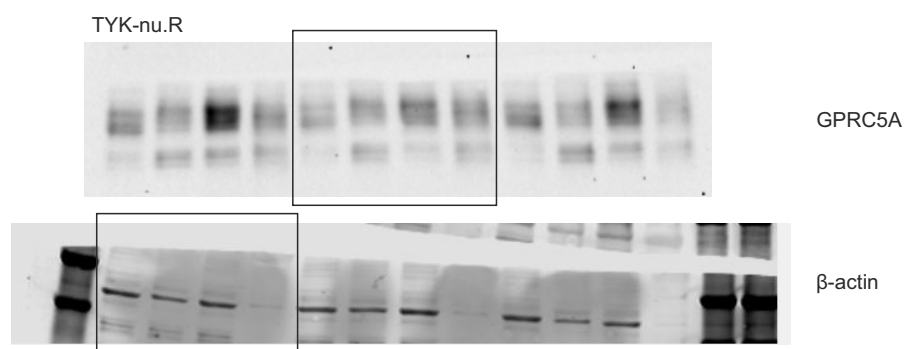

## D

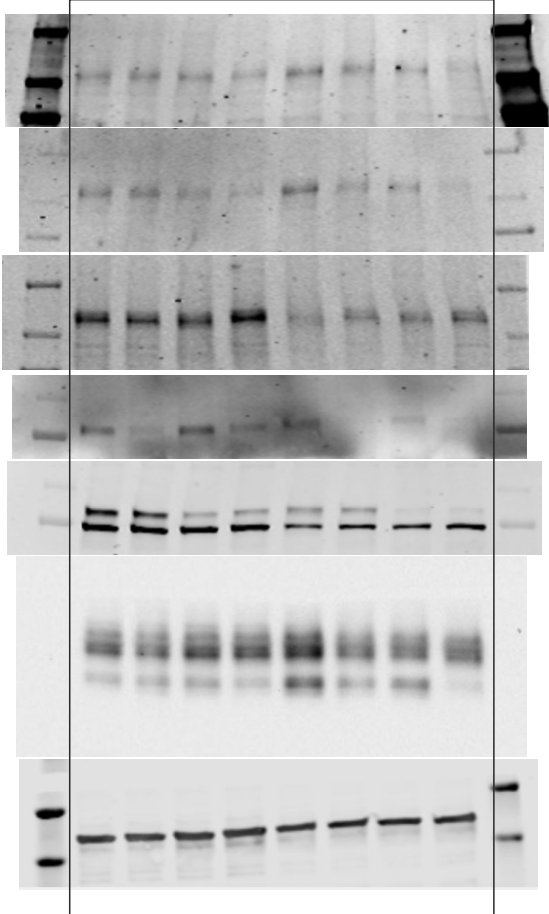

## E

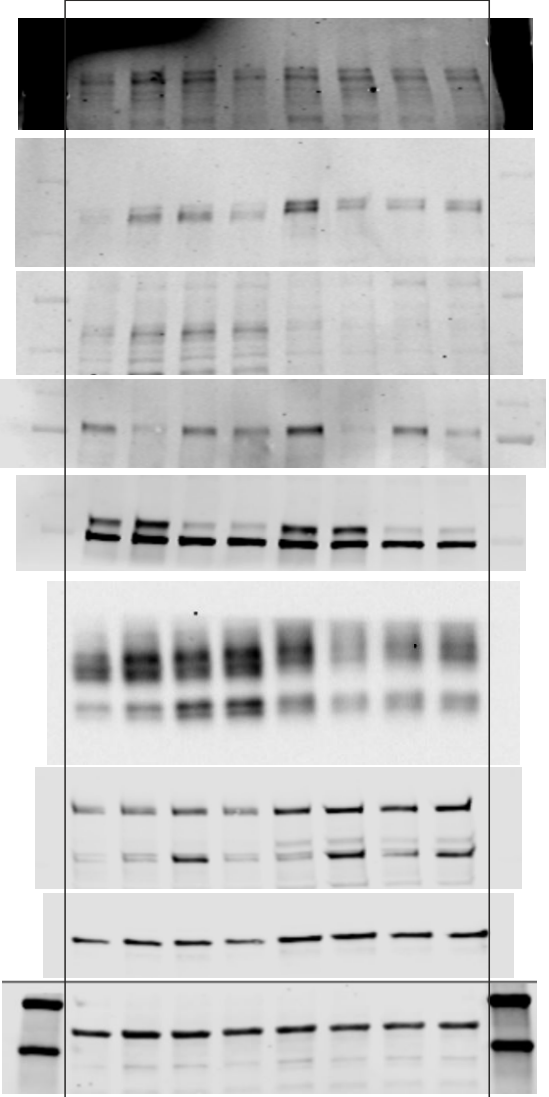

Supplement: Supplementary file 9 — Source Data for Figure 6 [file EMMM-12-e11177-s007.pdf]
