# Supplementary material for: Adaptive RSK‐EphA2‐GPRC5A signaling switch triggers chemotherapy resistance in ovarian cancer
Source: EMBO Mol Med. 2020 Mar 2;12(4):e11177. doi: 10.15252/emmm.201911177 (PMC7136956; doi:10.15252/emmm.201911177)
Supplement: Supplementary file 6 — Source Data for Figure 3 [file EMMM-12-e11177-s004.pdf]

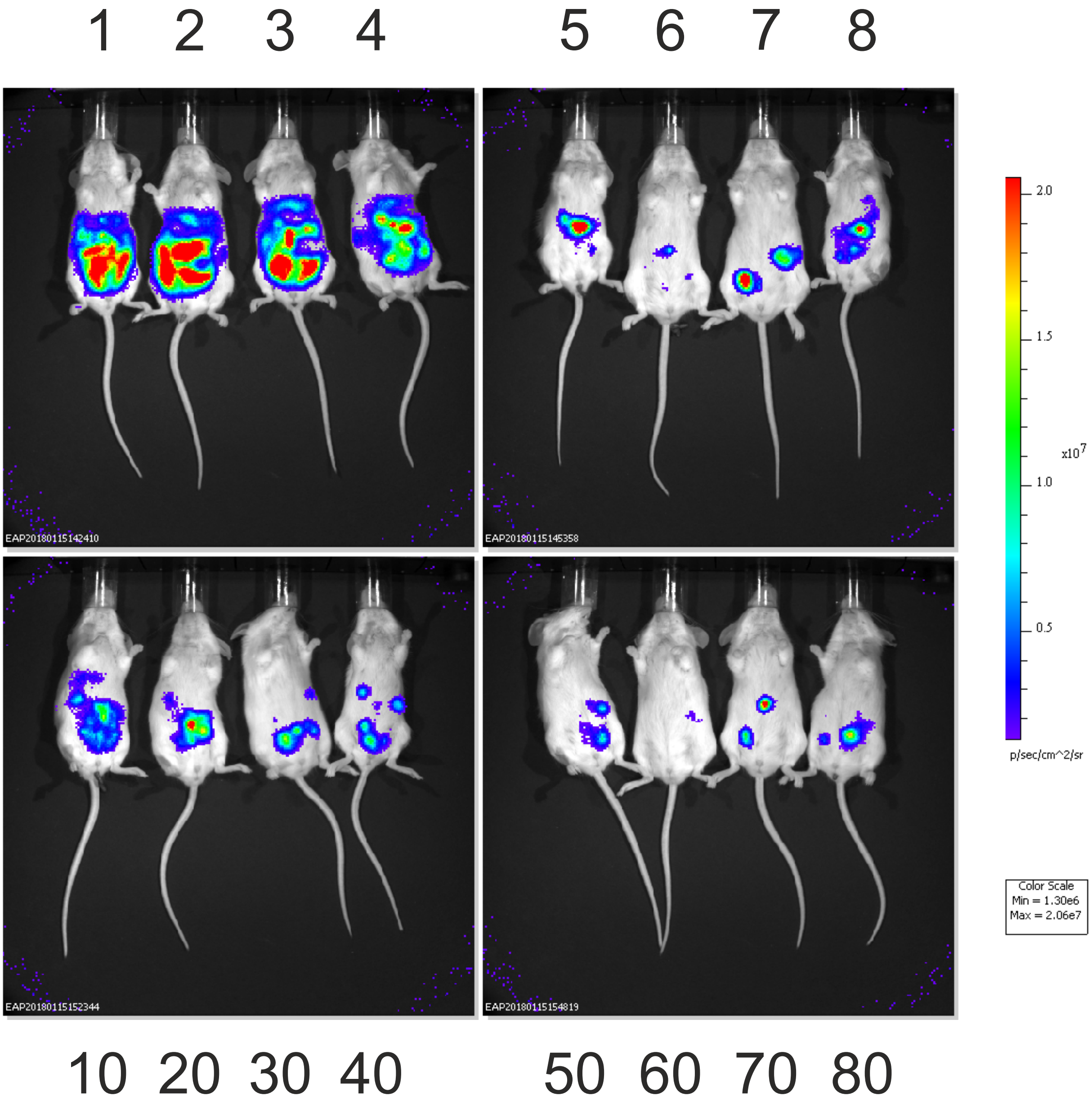

Control: 1, 2, 3, 4, 10
Carboplatin: 6, 30, 50, 70, 80

Carboplatin+BI-D1870 (not included in manuscript due to liver toxicity, forcing us to quit the dosing): 5, 7, 8, 20, 40, 60
