# Supplementary material for: Adaptive RSK‐EphA2‐GPRC5A signaling switch triggers chemotherapy resistance in ovarian cancer
Source: EMBO Mol Med. 2020 Mar 2;12(4):e11177. doi: 10.15252/emmm.201911177 (PMC7136956; doi:10.15252/emmm.201911177)

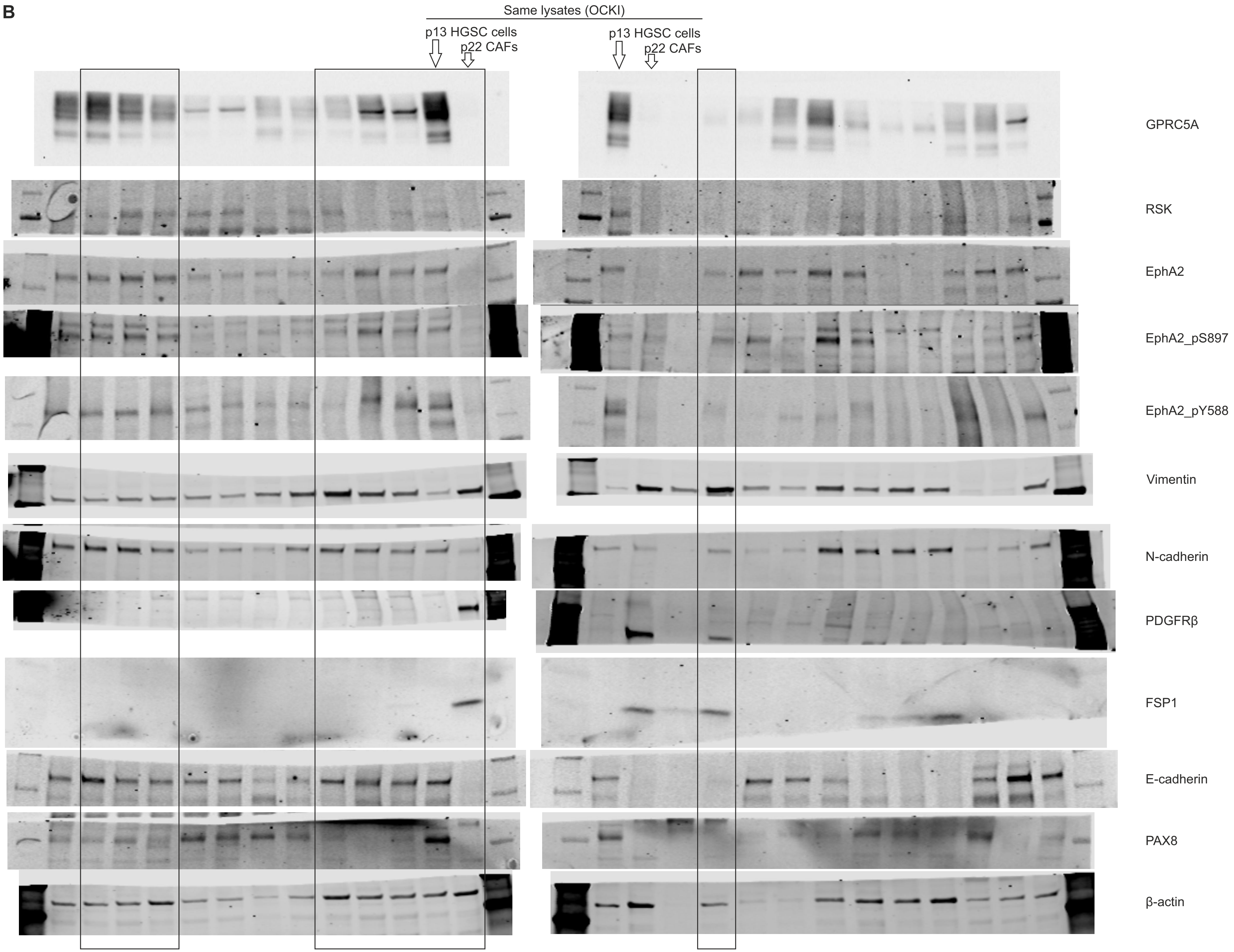
B
Same lysates (OCKI)
p13 HGSC cells
p22 CAFs
p13 HGSC cells
p22 CAFs
GPRC5A
RSK
EphA2
EphA2_pS897
EphA2_pY588
Vimentin
N-cadherin
PDGFRβ
FSP1
E-cadherin
PAX8
β-actin

C

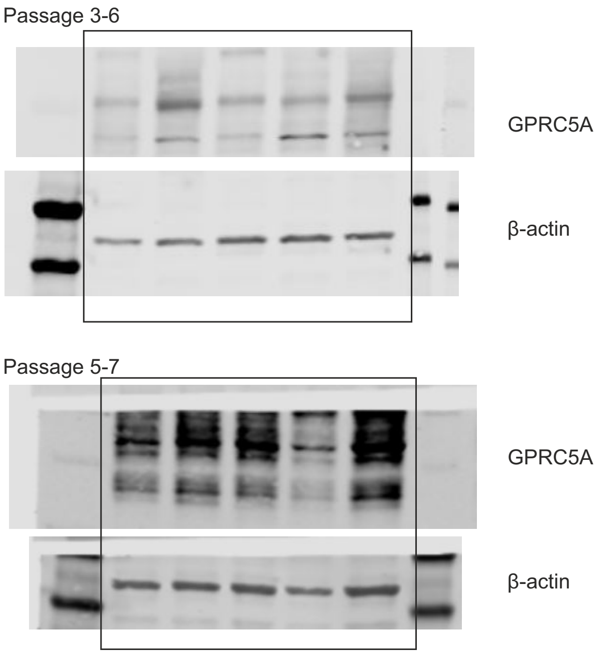

Supplement: Supplementary file 10 — Source Data for Figure 8 [file EMMM-12-e11177-s008.pdf]
